# Polymeric Heart Valves: Do They Represent a Reliable Alternative to Current Prosthetic Devices?

**DOI:** 10.3390/polym17050557

**Published:** 2025-02-20

**Authors:** Martina Todesco, Gianluca Lezziero, Gino Gerosa, Andrea Bagno

**Affiliations:** 1 Department of Industrial Engineering, University of Padua, 35131 Padua, Italy; martina.todesco.2@phd.unipd.it (M.T.); gianluca.lezziero@studenti.unipd.it (G.L.); 2 Department of Cardiac, Thoracic Vascular Sciences and Public Health, University of Padua, 35128 Padua, Italy; gino.gerosa@unipd.it

**Keywords:** polymeric heart valves, polymers, heart valve prosthesis, in vitro tests, valve replacement, in vivo trials

## Abstract

With the increasing number of people suffering from heart valve diseases (e.g., stenosis and/or insufficiency), the attention paid to prosthetic heart valves has grown significantly. Developing a prosthetic device that fully replaces the functionality of the native valve remains a huge challenge. Polymeric heart valves (PHVs) represent an appealing option, offering the potential to combine the robustness of mechanical valves with the enhanced biocompatibility of bioprosthetic ones. Over the years, novel biomaterials (such as promising new polymers and nanocomposites) and innovative designs have been explored for possible applications in manufacturing PHVs. This work provides a comprehensive overview of PHVs’ evolution in terms of materials, design, and fabrication techniques, including in vitro and in vivo studies. Moreover, it addresses the drawbacks associated with PHV implementation, such as their limited biocompatibility and propensity for sudden failure in vivo. Future directions for further development are presented. Notably, PHVs can be particularly relevant for transcatheter application, the most recent minimally invasive approach for heart valve replacement. Despite current challenges, PHVs represent a promising area of research with the potential to revolutionize the treatment of heart valve diseases, offering more durable and less invasive solutions for patients.

## 1. Introduction

Valvular heart diseases (VHDs) remain significant global health concerns, affecting approximately 41 million people worldwide and causing premature mortality, disability, and reduced quality of life [1]. As the population increases and ages, the prevalence of valve-related disorders (e.g., aortic stenosis or mitral regurgitation) continues to rise, leading to a growing demand for effective and durable heart valve substitutes. According to a report dated 2024, the global prevalence of VHDs for aortic and mitral valves is 13.3% and 15.5%, respectively [2]. Between 1990 and 2019, the incidence of calcific aortic valve disease, a major cause of heart failure, increased by 90% [2].

Currently, the primary treatment for VHDs is surgical valve replacement with either biological or mechanical valve prostheses, performed on approximately 250,000–300,000 patients worldwide per year [3]. Both mechanical and biological prosthetic devices suffer from severe limitations. On the one hand, mechanical valves impose lifelong administration of anticoagulant therapy due to the inherent thrombogenic nature of blood-contacting surfaces. This therapy heavily impacts patients’ quality of life and even safety: anticoagulants must be accurately dosed, and patients need to periodically check their coagulation level to avoid the risks of both bleeding and thrombotic events. Obviously, in the case of other kinds of surgery (e.g., dental surgery) and in the case of pregnancy, they have to suspend the anticoagulation therapy. On the other hand, biological prosthetic valves are prone to structural deterioration and calcification, which limit their durability.

The decision of which type of valve (mechanical or biological) should be implanted is mainly based on patient-related factors (e.g., contraindications to anticoagulation therapy, age, and place of residence).

In recent years, another type of prosthetic heart valve made of polymeric material has emerged as an alternative to both biological and mechanical devices, offering potential advantages in terms of durability, biocompatibility, and ease of manufacturing. Polymers represent a versatile class of materials, renowned for their favorable mechanical and physicochemical properties. Their key strengths lie in their cost-effective production, ease of fabrication, and the adaptability of their structure. These features enable the enhancement of biocompatibility, hemocompatibility, and material integration within the biological environment [4].

Polymeric heart valves (PHVs) are designed to overcome the drawbacks of current prostheses by eliminating animal-derived proteins and reducing the risk of calcification and immune response [5]. Moreover, advancements in polymer science and materials engineering have enabled the development of novel polymeric materials with tailored properties, such as enhanced flexibility, strength, and wear resistance.

This paper aims to explore recent developments in PHV technology, highlighting material selection, manufacturing techniques, design, and functional characteristics. By addressing the critical challenges associated with heart valve replacement, including thrombogenicity, durability, and hemodynamic performance, PHVs hold the potential to revolutionize the field of cardiovascular implants.

### Methodological Approach and Scope

In using the query “polymeric [Title/Abstract] AND heart valves [Title/Abstract]” on PubMed and ScienceDirect and excluding articles related to “biodegradable polymers”, a total of 289 articles were identified, published between 1974 and February 2025. In total, 195 references were selected and cited in the text (Table 1).

**Figure 1:**
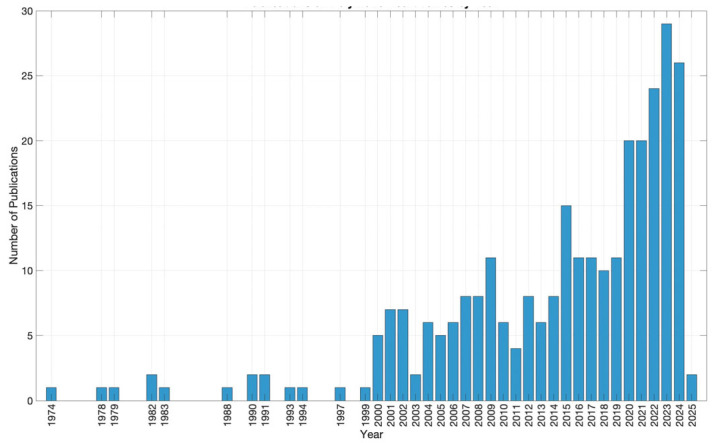
Number of publications related to polymeric heart valves found in PubMed and ScienceDirect from 1974 to February 2025.

The increasing number of publications over the years highlights the growing interest in the development of polymeric heart valves (Figure 1). This interest pertains not only to the creation of materials that ensure chemical stability, adequate mechanical properties, and resistance to calcification but also to interactions with the biological environment. In addition to biocompatibility, the materials have to also exhibit hemocompatibility, and the prosthetic heart valves must ensure optimal blood fluid dynamics.

The reported chemical structures were designed using Chemsketch Freeware software (Advanced Chemistry Development, Toronto, ON, Canada).

## 2. Native Heart Valves

**Figure 2:**
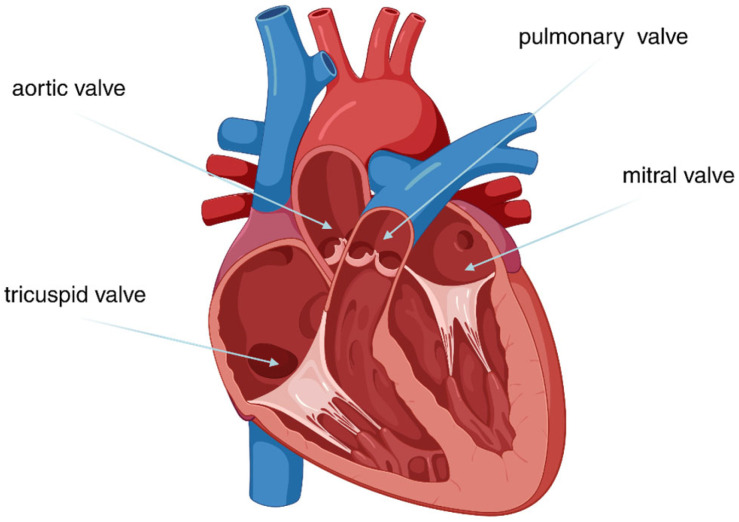
The four valves of the human heart. Created in BioRender. Bagno, A. (2025) https://BioRender.com/h95c965.

The heart contains four valves that regulate blood flow and move it in the correct direction, preventing backflow (Figure 2). The heart valves open and close cyclically in response to pressure changes [6]. The tricuspid and mitral atrioventricular valves separate the atria from the ventricles in the right and left sides of the heart, respectively. The aortic valve separates the left ventricle from the aorta, and the pulmonary valve separates the right ventricle from the pulmonary artery.

Heart valves are composed of flexible yet strong tissues, primarily made up of collagen, elastin, and glycosaminoglycans [6]. These structural components provide the mechanical properties needed for the valves to withstand repetitive opening and closing movements during each cardiac cycle.

The mitral valve is composed of several fundamental components: the mitral annulus, the valve leaflets, the chordae tendineae, and the papillary muscles [7]. The mitral annulus is a fibrous ring that provides structural support to the valve, maintaining its shape and helping it function properly during the cardiac cycle.

The tricuspid valve is made up of three cusps: posterior, anterior, and septal. These leaflets are connected to the annulus fibrosus, which provides structural support to the valve. The chordae tendineae connect the valve leaflets to the papillary muscles within the right ventricle [7].

The pulmonary valve consists of three semilunar cusps: anterior, right, and left cusps. Each of these is inserted in a fibrous ring [8]. During ventricular systole, the pulmonary valve cusps open, allowing deoxygenated blood to flow into the pulmonary artery and then into the lungs for oxygenation; during diastole, the semilunar cusps close to prevent blood from flowing back into the right ventricle [8].

The aortic valve is composed of three semilunar cusps: right, left, and non-coronary cusps, inserted into a fibrous ring [8]. It is the valve most frequently affected by valvular diseases, which can be due to various factors. The aortic valve must withstand the highest pressure within the heart, which can exceed 120 mmHg.

Heart valve leaflets are structured to withstand the dynamic environment of the heart and are mainly composed of three distinct layers. The fibrosa is the external layer, the thickest one, and is made of a dense network of collagen type I fibers [9]. Functionally, this layer provides a robust structure to resist mechanical forces during the cardiac cycle, and it extends over the entire leaflet surface. With regard to the aortic valve, the ventricularis layer (or atrialis layer for the mitral and tricuspid valves) is the internal one and faces the left ventricle (or left atrium for the mitral valve and right atrium for the tricuspid valve). It is composed of a dense network of elastin and collagen fibers [9]. The physiological function of this layer is to help in minimizing large radial strains that could appear when the valve is fully opened and the forward flow is maximum [9]. In the middle, there is the spongiosa layer, which contains a high concentration of glycosaminoglycans (GAGs) and proteoglycans (PGs) [10]. This layer imparts flexibility and resilience to the valve leaflets and helps maintain hydration and lubrication, crucial properties for durability and ability to withstand the repetitive stresses [10]. All these layers are encased in a sheath of endocardial endothelial cells interlaced with valve interstitial cells [6]. These cells have homeostatic activity that aids in the daily function of the valve. They create a barrier that avoids the adhesion of blood cells and platelets, preventing blood clot formation [10]. Moreover, this layer adapts to mechanical stresses imposed by the pulsatile nature of the bloodstream, plays a role in modulating local inflammation (e.g., endothelial cells can release cytokines and chemokines in response to injury or inflammation), and can produce and release various molecules that regulate blood vessel tone and blood flow dynamics (e.g., endothelial cells can release nitric oxide (NO), which helps ensure optimal blood vessel dilation) [6,9].

The mechanical and biological properties of native heart valves, resulting from their complex structure, form the basis for research and development in the field of polymeric heart valves.

## 3. Valvular Diseases

Native heart valve diseases (also known as valvulopathies) encompass a range of conditions that impair valve function, leading to significant cardiovascular complications. These diseases can be due to congenital defects, degenerative changes, inflammatory processes, or infectious agents. The main pathologies and their clinical manifestations are briefly mentioned herein:

- Rheumatic Heart Disease (RHD): It is a chronic condition that mainly affects children and young adults in developing countries, where access to adequate healthcare and antibiotic treatments is limited [11]. RHD results from acute rheumatic fever (ARF) [12]. If not treated, repeated infections can occur, leading to long-term complications and increased morbidity and mortality [12,13].
- Infective Endocarditis (IE): It is a severe microbial infection that can affect the inner layer of the heart chambers and valves or indwelling cardiac devices [14], causing a high mortality rate: about 19–82% for 5-year mortality and 6–50% for in-hospital mortality [14]. The main risk factors for developing IE include valve replacement, cardiac factors (e.g., congenital valvular abnormalities, rheumatic heart disease, degenerative valvular disease, or post-cardiac implant of cardiac devices), and non-cardiac risks (e.g., intravenous drug use, chronic liver disease, hemodialysis, poor dentition, or advanced age) [15].
- Heart Valve Prolapse: It is a condition in which one leaflet bulges or prolapses backward during contraction. This typically affects the mitral valve, but rarely, it can also involve other valves. Prolapse means that the valve does not close properly, resulting in valve regurgitation, complicated by heart failure or arrhythmia, that could also lead to sudden death [16]. Mitral valve prolapse (MVP) occurs in about 2–3% of the general population [17].

**Figure 3:**
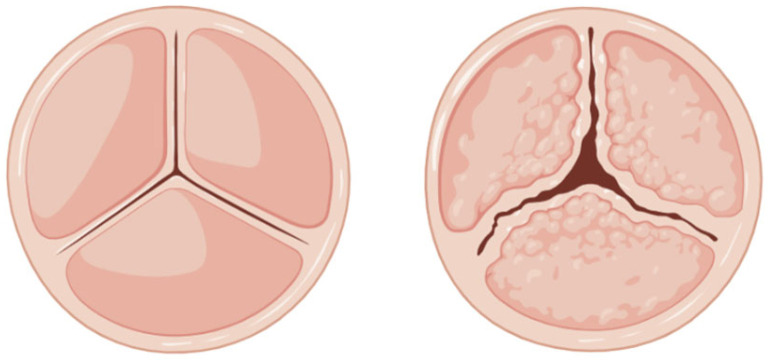
(**Left**): normal aortic valve. (**Right**): the presence of calcific deposits on the leaflets causes stenosis. Created in BioRender. Bagno, A. (2025) https://BioRender.com/t72l697.

Clinical manifestations of valvulopathies primarily include heart valve stenosis and regurgitation. Stenosis is a condition characterized by the narrowing of one or more native heart valves (mostly impacting the aortic and mitral valves), which restricts blood flow. There are several causes of valve stenosis: inflammatory rheumatic heart disease, degenerative calcification, congenital heart defects (e.g., congenitally bicuspid aortic valve), endocarditis, and radiation therapy [18]. Pulmonary valve stenosis is almost always congenital in origin [19]. The main causes of aortic stenosis are rheumatic valve disease, calcific degeneration (Figure 3), and congenitally bicuspid aortic valve (often with superimposed calcific changes). Early detection and appropriate management are crucial to prevent the progression of the disease and maintain optimal heart function.

Heart valve regurgitation (also known as valve insufficiency) occurs when a heart valve fails to close properly, allowing blood to flow backward. This condition can affect each of the four heart valves, but it is more common for the mitral and aortic ones. Aortic regurgitation can mainly be due to a bicuspid valve or aortic root dilation [20]. Mitral regurgitation is also fairly common in the population, and for people aged 75 or more, it occurs in around 10% [21]. In general, causes of valve regurgitation include degeneration, inflammation, tissue disruption, infection, trauma, iatrogenic factors, or congenital abnormalities [22].

## 4. Current Therapeutic Approaches

Various therapeutic approaches for heart valve diseases exist, including pharmacological treatments, minimally invasive procedures, and surgical interventions aimed at repairing or replacing diseased valves. While surgical interventions are frequently necessary, they are often considered the last option due to their invasive nature and associated risks.

### 4.1. Pharmacological Approaches

Pharmacological treatments depend on the type and severity of the disease. Drugs may be useful in cases of less severe valvular diseases or as a bridge to surgery for patients with more severe diseases. For aortic stenosis, studies have been conducted with statins, bisphosphonates, and ACEIs/ARBs [23]. Statins have not shown significant effectiveness in slowing disease progression or improving clinical outcomes [15]. Similarly, since bisphosphonates can inhibit vascular calcification, different studies investigated their potential to delay progression or induce the regression of aortic stenosis, but with no success [15]. Enzyme inhibitors (ACEIs) and angiotensin receptor blockers (ARBs) have shown reduced calcium accumulation during studies, but they need further investigations regarding stenosis. For aortic regurgitation, the focus is on different vasodilators and beta-blockers [15]. For mitral regurgitation (MR), ACEIs/ARBs are commonly employed for both types but are more useful in functional MR, while beta-blockers are currently under study, but with no beneficial results [15]. Regarding mitral stenosis, beta-blockers or non-dihydropyridine calcium channel blockers can attenuate symptoms and correct hemodynamic abnormalities by reducing heart rate to lengthen diastole [15].

To prevent or combat infectious diseases, antibiotics are prescribed, especially during dental procedures or other surgeries [24]. Regarding RHD, penicillin is commonly used, while macrolide antibiotics (e.g., erythromycin, roxithromycin, or azithromycin) are preferred for patients with allergic reactions to penicillin [25]. Recently, other antibiotics were approved by the Food and Drug Administration (FDA) and the European Medicines Agency (EMA), such as oritavancin, dalbavancin, ceftaroline, and ceftobiprole, showing a good outlook for treating various infections [26].

Another pharmacological treatment is based on antithrombotic therapy, including anticoagulant and antiplatelet drugs. This is an important treatment, particularly for the following: prosthetic heart valve recipients, for whom the risk of thromboembolic complications is greatest during the first three months after surgery; patients undergoing valve repair; and those with specific native valve conditions that predispose them to thromboembolic events [27]. Commonly used anticoagulants are vitamin K antagonists (VKAs) and direct oral anticoagulants (DOACs) [26]. VKAs inhibit the synthesis of vitamin K-dependent clotting factors; warfarin is the most used, often for life-long anticoagulation therapy following mechanical device implantation [27]. DOAC are used to prevent stroke in atrial fibrillation, but they are generally not recommended for patients with prosthetic heart valves due to the higher risk of thromboembolic events and valve thrombosis compared to warfarin [27]. Antiplatelet agents are often used in combination with anticoagulants: aspirin is the most used, inhibiting platelet aggregation [28].

### 4.2. Surgical Approaches

The surgical approaches for treating heart valve diseases are usually categorized into two groups [29], standard (conventional) and device-based, with the choice depending on the specific valve disease, patients’ health, and risk factors. Standard surgery requires full sternotomy, cardiopulmonary bypass (CPB), and cardioplegic arrest (CA). The catheter-based approach is much less invasive: it is performed through the transcatheter implantation of aortic and mitral valves in a beating heart. The main procedures are listed herein:

- Mitral valve Transcatheter Edge-to-Edge Repair (M-TEER): It is a non-surgical treatment mainly for mitral regurgitation; it is suitable for patients who are not candidates for open-heart surgery. The M-TEER procedure involves a device to clip together the valve leaflets to reduce regurgitation [28]. Specifically, a guide catheter is used in the percutaneous procedure, which is inserted typically from the right femoral vein into the left atrium. A device with a clip is then steered through the mitral valve and monitored (e.g., by echocardiography and fluoroscopy) until the mitral regurgitation is reduced [29].
- Percutaneous Balloon Valvuloplasty (PBV): It is a minimally invasive procedure used to treat stenotic heart valves and aims to restore its normal functionality [30,31]. The procedure involves the insertion of a guidewire through a vascular access site to the stenotic valve, threading a balloon catheter over the guidewire, and carefully positioning it across the stenotic valve [28]. The balloon is then inflated up to a specific pressure to dilate the valve. Once the valve has been adequately dilated, the balloon is deflated and removed [28].
- Transcatheter Valve Replacement: It is a minimally invasive procedure used to replace a diseased heart valve without the need for open-heart surgery. This procedure can be used to treat both aortic and mitral valves. Vascular access is typically performed through the femoral artery (transfemoral approach), although other access points may be used if the femoral route is unsuitable [32]. A catheter is inserted through the access site and guided to the heart under imaging monitoring. For transcatheter aortic valve replacement (TAVR), a crimped valve mounted on a balloon-expandable or self-expanding stent is advanced through the catheter to the diseased valve. Once in the correct position, the new valve is deployed either by inflating the balloon or allowing the stent to self-expand, pushing the diseased valve leaflets aside and anchoring the new valve in place [33]. For transcatheter mitral valve replacement (TMVR), a similar approach is used, but navigation and deployment can be more complex due to the anatomical position of the native valve [34]. This type of valve replacement offers better results and might be technically less challenging than other surgical procedures. Nevertheless, it may come with a higher number of complications, and long-term follow-up data are limited [32].

Regarding traditional surgical interventions (repair and replacement), they involve open-heart surgery and remain crucial for complex cases or when less invasive procedures are not suitable or have failed.

When possible, valve repair is preferred over replacement, as it preserves the patient’s own valve and often results in better heart function and a lower risk of complications. Procedures can concern different parts of the valve. Leaflet repair implies fixing the valve leaflets by removing or adding tissue to ensure their proper closure. It is often used to treat valve prolapse, deformities, or minor lesions [35]. These procedures include the resection of prolapsed segments, stitching torn leaflets, and transferring or reattaching the chordae tendineae [33]. Another procedure is annuloplasty, where the valve annulus is reinforced to tighten or reshape it, leading to the improved coaptation of the leaflets and a narrowed valvular orifice [33]. This can be realized using different techniques, such as simple sutures, posterior annuloplasty bands, or prosthetic rings. To treat stenosis, mainly in the mitral valve, commissurotomy is required: it consists of cutting fused valve leaflets to widen the valve opening [36]. For the aortic valve, different repair techniques are employed: commissuroplasty with Cabrol sutures, root remodeling, root reimplantation, and plication are the most common procedures [37].

If the valve is too damaged to be repaired, replacement is necessary. This procedure involves the surgical removal of the native valve and implantation of a valve prosthesis. The new valve can be of different types, each with specific features, advantages and drawbacks. The choice of device depends on various factors, such as the patient’s age and health conditions and the specific valve that needs to be replaced.

## 5. Prosthetic Heart Valves

Prosthetic heart valves currently used in cardiac surgery are mechanical and bioprosthetic. Polymeric heart valves are still undergoing several studies and investigations due to their favorable properties and promising prospective, but currently, there are very few clinical trials. Other types of valve substitutes are allograft and autografts: they are fully biological prostheses, which do not require any chemical fixation.

- Allografts: They are the valves withdrawn from human donors (cadavers), exhibiting several advantages. They mold well to the infected aortic annulus, have good resistance to infection compared to synthetic materials, do not require anticoagulation, and have excellent hemodynamic performances (especially for small sizes) [38]. This makes them particularly useful in cases of infection, such as IE. Allografts are commonly used for aortic valve replacement and are especially valuable for patients with complex valve infections or those undergoing reoperative procedures. Several studies have proven that allograft replacements can significantly improve long-term clinical outcomes, but the lack of donors and possible incompatibilities remain issues [38,39].
- Autografts: The Ross procedure implies the use of the patient’s own pulmonary valve to replace the diseased aortic valve. The pulmonary valve is then replaced with an allograft. This procedure offers more advantages than allografts, such as the potential for use in pediatric patients due to its propensity for repair and growth, the internal innervation of the cusps, and higher cellular viability [38,40]. However, the increased complexity and longer duration of surgery complicate the replacement procedure [38]. Several studies have shown long-term outcomes for patients, but the procedure is still characterized by limited suitability and possible complications [39].
- Mechanical Heart Valves (MHVs): They are made of synthetic materials, such as titanium and pyrolytic carbon. On average, they can function for up to 25 years due to their high durability, reducing the need for revision surgeries [41]. On the other hand, since they are made of non-biological materials, they require lifelong anticoagulation therapy to prevent thromboembolic events [40]. Since the 1960s, different types of valve designs have been developed: from the caged ball [42] and non-tilting disk valves [43] to the more recent tilting disk valves [39,42]. Presently, the most widely used MHVs are the bileaflet ones, which have two oscillating leaflets that open and close to regulate blood flow [41]. Since the St. Jude valve was introduced in 1977, more than 600,000 have been implanted; currently, they are the most implanted MHVs due to their reliability and efficient blood flow dynamics [39]. Despite the durability of MHVs, they present several drawbacks. Blood flow around the valves causes high shear stress, which can trigger platelet activation, leading to clot formation on the valve surface and increasing the chance of thromboembolic effects [41]. The use of lifelong anticoagulation therapy (e.g., warfarin) reduces the risk but increases the hemorrhagic probability. Other factors that hinder the use of MHVs include the presence of other diseases, such as thrombotic diseases or atrial fibrillation [41].
- Bioprosthetic Heart Valves (BHVs): They are made of animal tissues, typically bovine pericardium or porcine heart valves, after chemical treatment with glutaraldehyde, which sterilizes the tissues and makes them immunologically compatible with the recipients, avoiding any adverse event [44]. Unlike MHVs, BHVs do not require lifelong anticoagulation therapy, reducing the risk of bleeding. Additionally, they offer good hemodynamic performance similar to native valves and do not produce any audible noise, which can be a comfort factor for some patients [41]. On the other hand, they have a shorter lifespan than MHVs (typically 10–20 years), mainly due to calcification and degradation, making them not suitable for younger patients [41,44,45]. Annually, tens of thousands of BHVs are implanted in elderly patients, but the number of younger people with heart valve diseases is growing [42].

## 6. Polymeric Heart Valves

Polymeric heart valves (PHVs) offer an appealing alternative to current heart valve prostheses, as they can address significant limitations of both MHVs and BHVs. These devices mimic the natural functioning of native heart valve leaflets while also being conducive to large-scale production.

A complete explanation and assessment of PHVs can be presented according to a variety of criteria. They will be discussed based on the specific polymers employed for their production, the geometry used in leaflet design, manufacturing techniques, and possible causes of failure.

### 6.1. The First Generation of Polymers for PHVs

#### 6.1.1. Polysiloxanes

**Figure 4:**
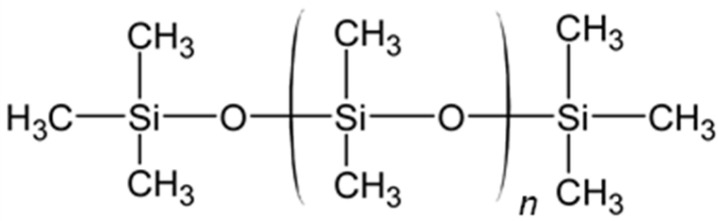
Chemical structure of polydimethylsiloxane (PDMS): the backbone is characterized by the alternation of Si and O atoms.

The first polymers used in the concept of PHVs were polysiloxanes, commonly referred to as silicones. Polysiloxanes are characterized by alternating silicon and oxygen atoms in the molecular backbone, along with the capability of accommodating various pendant groups attached to the silicon atom. The most common form, with the repetition of the monomer [Si(CH_3_)_2_O], is polydimethylsiloxane (PDMS) (Figure 4).

The physical properties of silicones are influenced by the average length and the degree of cross-linking among polymer chains [46]. The strong Si-O bond contributes to the high bond strength of silicones, which in turn provides them with thermal and chemical stability; additionally, biocompatibility, bio-durability, and fatigue resistance make them versatile materials for biomedical applications [46,47].

One of the first flexible trileaflet valves made of silicone was developed in the 1950s by Roe et al. and tested in animals [48]. Evaluated in an ascending aorta model of a dog, these valves exhibited satisfactory functions in the initial stage. However, the first clinical study was unsuccessful because of post-surgery blood clot complications and high mortality rate [49]. Despite the initial failure, several endeavors to create polymeric heart valves using silicone elastomers have been made. In the 1960s, Roe et al. produced a tricuspid aortic prosthesis from Dacron and polysiloxane, which was implanted in 18 patients [50]. A limited clinical trial was conducted between 1960 and 1962; however, it was discontinued due to the high mortality rate [51]. Most of the deaths occurred due to clinical complications despite the correct functioning of the leaflet mechanism; some embolic complications were also observed in three patients. Follow-up studies reported that the prostheses continued to function properly for 33 to 61 months after implantation [51].

Hufnagel et al. developed and tested trileaflet PHVs fabricated with a composite silicone rubber–polypropylene fabric and implanted in 20 patients [52]. A study was conducted to examine necropsies from these patients who underwent aortic valve replacement. The study concluded that although the trileaflet aortic prosthesis resembles the normal aortic valve in design, these materials are not durable enough to withstand the hemodynamic stresses in this position [52].

Mohri et al. revisited and tested trileaflet silicone valves in the 1970s [53]. Their study examined the durability and performance of Silastic trileaflet aortic valves, highlighting concerns regarding water absorption and large-scale production. It also explored the importance of vortex formation during the closing phase, which can be a way to prevent fibrin deposition and blood clot formation and identified potential thrombotic effects in animal trials. Regional heparinization was proposed as an effective solution [53].

Despite the extensive utilization of polydimethylsiloxane-based devices and implants across various medical disciplines, these early studies showed that durability was a critical issue that did not keep up with the pressure generated by blood flow. Other studies, even recently, demonstrated positive outcomes with the use of PDMS, either as a coating, a surface-modifying end group, or when incorporated into other polymers.

In the early 2000s, Simmons et al. illustrated the efficacy of a flexible PDMS-based polyurethane, synthesized using 20% poly(hexamethylene oxide) (PHMO) and 80% PDMS macrodiols, which exhibited excellent biostability and improved long-term biocompatibility. The functional properties were determined by both in vitro and in vivo tests, using scanning electron microscopy (SEM) to investigate explanted samples from sheep after 24 months [54]. Additionally, PDMS proved to be beneficial when employed as a surface modifier for polyurethane. Due to the low surface energy, siloxane groups migrate onto the surface, creating a siloxane-rich polyurethane surface [55]. This modification was shown to provide enhanced in vivo degradation resistance. However, under high-strain conditions, it revealed pitting and cracking damages [56]. Dabagh et al. further investigated the effects of PDMS grafting onto polyurethane [57]. In a 30-day in vitro study, they observed that surface modification of polyurethane films prevented calcification and exhibited no cytotoxicity but did not alter platelet adhesion due to the good blood compatibility of both polymers.

To address the long-term durability of this material, interest in the combination of PDMS and polycarbonate urethane (PCU) has grown over the years. The durability and mechanical resistance of PCU complement the advantages of PDMS, which is flexible and biocompatible. A study on PDMS-PCU showed that PDMS tends to accumulate on the surface layer, protecting the bulk from degradation and enhancing oxidative resistance [58]. However, high PDMS fractions may result in possible residual stress, undesirable visual defects, or weak interfacial interactions. The optimal molar ratio of PCU/PDMS was found to be 90:10, which simultaneously improved oxidation and hydrolysis resistance while maintaining a proper mechanical performance [58]. This material can find potential use in long-term implantation applications.

Another recent study investigated the potential application of a biostable PDMS-based polyurethane–urea bearing zwitterion sulfobetaine (PDMS-SB-UU) [59]. The material was successfully synthesized, and its stability was confirmed by in vitro tests against 30% hydrogen peroxide and lipase for 8 weeks. It also demonstrated higher resistance to platelet adhesion and fibrinogen adsorption compared to PDMS. Additionally, PDMS-SB-UU exhibited no cytotoxicity with rat vascular smooth muscle cells or hemolysis with ovine blood [59]. PDMS-SB-UU can be utilized either as the entire matrix or as surface coating for several blood-contacting medical devices, such as prosthetic heart valves.

#### 6.1.2. Polytetrafluoroethylene

**Figure 5:**
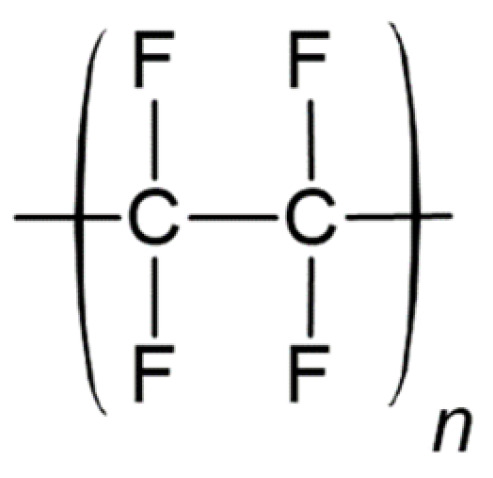
Chemical structure of polytetrafluoroethylene (PTFE).

Polytetrafluoroethylene (PTFE) (Figure 5) has another commonly used trade name, Teflon. It contains only carbon and fluorine atoms. PTFE exhibits high chemical inertia, high stability, and strong mechanical properties [60]. Due to these features, PTFE is used in various biomedical applications.

Braunwald and Morrow performed clinical trials with flexible tricuspid Teflon fabric valves, based on the Muller–Littlefield prosthesis [61]. They carried out hemodynamic assessments and postoperative clinical examinations of valve function in 23 patients who underwent the replacement of the aortic valve with flexible PTFE prosthesis. Left heart catheterizations indicated satisfactory valve function in 21 out of 23 patients. However, severe aortic regurgitation developed in 13 patients, leading to death or reoperation [62]. An examination of explanted Teflon valves revealed the stiffening and calcification of the fabric, causing leaflet tearing and valve regurgitation. The failure of Teflon aortic prosthesis was attributed to unsatisfactory material rather than improper design.

At the end of the 1960s, Robert W. Gore introduced a new iteration of the material, expanded polytetrafluoroethylene (ePTFE). ePTFE was patented in 1976 and known to the public as Gore-Tex; it is formed by mixing PTFE powder with lubricant to create a paste, which is extruded into a film or tape, and then the lubricant is removed by heating, and the result is stretched at high temperature [63]. ePTFE is widely employed in biomedical applications due to its mechanical, chemical, and biocompatibility properties [63].

One of the earliest applications of Teflon heart valves dates to the late 1980s, when 12 prostheses were implanted in the tricuspid position in a sheep model [64]. In half of the valves, large calcific deposits were found on the inflow surfaces near the stent posts.

The application of ePTFE valves is currently limited. Medical devices made of ePTFE are not frequently used due to the risk of dysfunction caused by the thickening, calcification, or constriction of the valves, alongside suboptimal hemodynamic performance.

#### 6.1.3. Polyurethanes

**Figure 6:**
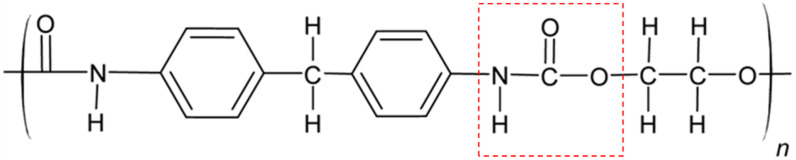
Chemical structure of a typical polyurethane (PU): the dotted box evidences the polyurethane linkage.

Polyurethanes (PUs) are a large group of polymers potentially suitable for the development of prosthetic heart valves (Figure 6). They are distinguished by the presence of a urethane linkage and can be synthesized through an addition reaction between an alcohol and an isocyanate. PUs are extensively studied due to their convenient synthesis, achievable at room temperature and under mild conditions [65]. Polyurethanes are widely used in cardiovascular applications due to advantageous mechanical and physiochemical properties. Moreover, some of them are also characterized by high biocompatibility that allows their unrestricted use in blood-contacting devices [66]. Furthermore, the Pus’ surface exhibits excellent resistance to microbial contamination, while thrombi formation is nearly comparable to PTFE.

Braunwald et al. designed and fabricated a prosthetic valve for mitral position made of flexible polyurethane, with the leaflets controlled via Teflon-made chordae tendineae [67]. In 1959, they implanted these valves in 27 dogs. Despite some difficulties during the experiment, the prosthetic valve was ultimately deemed satisfactory. In 1960, the same valves were used for complete mitral valve replacement in two humans [68]: one patient survived the procedure but died, presumably of an arrhythmia, four months later.

In the following years, many valves made of different PUs were tested both in vitro and in vivo. In 1988, an animal test was carried out to assess different valves made of different PUs [69]. Two valves from each material were implanted in the mitral position of Jersey calves. The survival times ranged between 127 and 291 days. While all explanted valves showed calcification and obstruction, these findings suggested that at least two PUs achieved survival times significantly surpassing those of bioprostheses under similar implant conditions.

In 1991, Jansen et al. introduced a novel three-leaflet valve, the J-3, designed to mitigate membrane stress [70]. The J-3 valve demonstrated superior hydrodynamic performance, thereby minimizing the risk of thrombus formation. In vitro testing revealed excellent durability and minimal shear stresses in the downstream flow field. However, animal tests showed poor resistance to calcification [70]. In 1996, a new PU trileaflet valve was developed, consisting of three thin polyurethane leaflets suspended within a flexible polyurethane frame [71]. The valve showed good results in hydrodynamic tests and exhibited good durability, with an equivalent lifespan of 10 years.

Bernacca et al. manufactured and evaluated several prosthetic heart valves. In 1996, they examined prosthetic heart valves made of polyetherurethane (PEU) [72]. These valves showed minimal calcification, particularly in regions of material failure, whereas bovine pericardium or porcine aortic valve biomaterials demonstrated much greater calcification under the same conditions. In 1997, they tested prosthetic heart valves made of polyetherurethaneurea (PEUE) [73]. These valves demonstrated superior mechanical performance, particularly in terms of fatigue resistance, compared to PEU valves. However, no significant improvement was observed concerning calcification.

In 2003 and then in 2004, a new PCU heart valve was introduced for both mitral and aortic positions, with the trade name ADIAM [74]. PCU contains hard and soft segments, for which their ratio determines material hardness. Both prostheses were designed to mimic physiological flow. The ADIAM mitral valve showed no material degradation, intrinsic calcification, or other signs of structural degradation, under neither in vivo nor in vitro settings. Additionally, no thromboembolic complications were observed [74]. The ADIAM aortic valve demonstrated in vivo durability with no increased thromboembolic complications and no intrinsic calcification. However, in vivo tests on animals had some limitations due to anatomical reasons, as they increased or caused some of the above-mentioned complications [74]. Both prostheses have not reached the clinical trial phase.

Although PUs exhibit satisfactory mechanical properties, their limited biocompatibility and biostability hinder the development of an optimal PHV.

### 6.2. Novel Polymers for Prosthetic Heart Valves

Several recently developed polymers, differing in composition and structure from those previously described, are currently available for the production of PHVs. These new materials offer enhanced mechanical properties and improved biocompatibility.

#### 6.2.1. POSS-PCU

**Figure 7:**
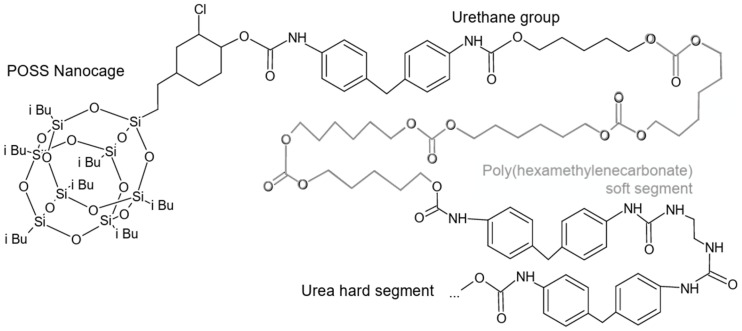
Chemical structure of POSS-PCU: the polyhedral oligomeric silsesquioxane (POSS) nanocage is incorporated into poly(carbonate-urea) urethane (PCU) with alternating soft and hard segments.

Polyhedral oligomeric silsesquioxane poly(carbonate-urea) urethane (POSS-PCU) is a nanocomposite polymer with superior biostability and biocompatibility compared to previous materials. These enhancements are achieved by improving the high resistance of PCU to cyclic loads through the creation of composites based on polyhedral oligomeric silsesquioxane (POSS) nanoparticles (Figure 7). It is a non-toxic material that possesses excellent thromboresistance and resistance to degradation [75,76]. It was demonstrated that the presence of the POSS group protects the soft segment of the PU, which is responsible for its flexibility and elasticity, from oxidative and hydrolytic degradation [76]. Kannan et al. compared POSS-PCU with PTFE and PCU and found that POSS-PCU has a greater thromboresistance than both PTFE and PCU with a lower absorption of fibrinogen, significant decrease in clot strength, and increase in clot lysis [75].

A first heart valve prototype made of POSS-PCU was developed at UCL (University College London, London, UK), possessing excellent mechanical strength, favorable surface properties, and resistance to platelet adhesion [77]. The nanocomposite was also utilized by Rahmani’s group for the development of a new semi-stented aortic valve (SSAV), optimizing the manufacturing process to enhance the precision and consistency required in heart valves by implementing an automated dipping and coating process [78]. The result was a polymeric heart valve with potential clinical application. The leaflet thickness played a fundamental role in the hydrodynamic performance: the thinnest valve (100 µm leaflet thickness) performed better than thicker valves or any bioprosthetic valve.

**Figure 8:**
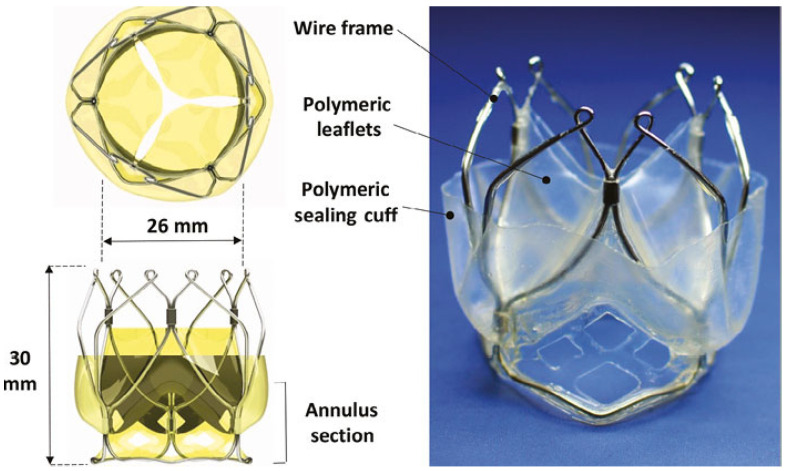
The TRISKELE heart valve. Reproduced with permission from [80], Europa Digital & Publishing, 2016.

The POSS-PCU also played a significant role in the development of transcatheter heart valves: in 2016, the Rahmani’s group developed a trileaflet valve, the TRISKELE valve (Figure 8) [79]. Utilizing new technologies to ensure repeatability and rapid production at lower costs, this valve has undergone both in vitro and in vivo tests. The in vitro study proved the excellent hydrodynamic performance of the TRISKELE valve, showing a significant reduction in paravalvular leakage, regardless of the presence of calcification [79]. In an in vivo study, the TRISKELE valve was successfully implanted in an acute preclinical ovine model with a 30 min postoperative follow-up. It exhibited optimal hemodynamic performance after catheter removal, although the presence of regurgitation was noted [80].

#### 6.2.2. FGO-PCU (Hastalex)

**Figure 9:**
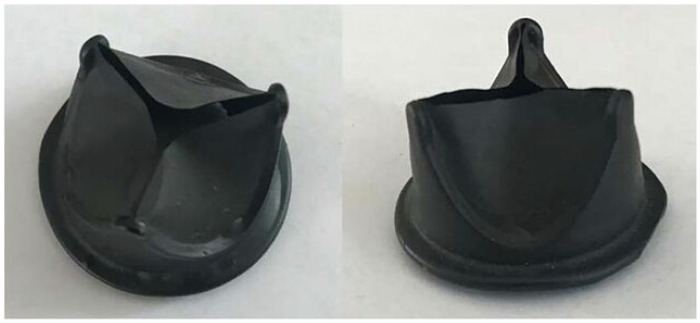
Prototype of the heart valve made from Hastalex. Reproduced from [81], Springer Nature, 2020.

Hastalex is a novel nanocomposite material based on the incorporation of functionalized graphene oxide (FGO) into the main chain of poly(carbonate-urea) urethane (PCU) developed by Ovcharenko et al. [81], who described in detail the synthesis of the Hastalex nanocomposite. The material has found several applications, for instance, as an antifouling agent in marine applications or as fibers in the textile industry. The initial tests performed by Ovcharenko’s group showed mechanical properties comparable with ePTFE, yet superior tensile strength that exceeds the load of native valve tissues and blood vessels. Although possessing a great capacity for elongation under extreme loads, it exhibits a relatively rigid nature within the range of physiological loads compared to common biological materials used for manufacturing prosthetic heart valves. Hastalex has a much higher Young’s modulus than ePTFE (11.3 MPa versus 1.9 MPa along the longitudinal direction, respectively), which is a crucial feature in minimizing energy loss [81]. The hydrodynamic performance of Hastalex heart valves depends on leaflet design, but the high deformability and higher rigidity of this material allow for the realization of thinner leaflets. Additionally, Hastalex is a hydrophobic material thanks to the presence of the functionalized graphene oxide. In vitro tests showed that Hastalex has no cytotoxic effect, but it is able to promote high cell adhesion, viability, and proliferation. Moreover, in vivo testing on rats showed that Hastalex possesses superior resistance to calcification when compared to ePTFE and cattle-derived pericardium. Currently, only one prototype of a Hastalex-based polymer heart valve has been developed (Figure 9): despite the promising advantages in cardiac surgery applications, more in vitro and in vivo tests are needed before moving toward clinical trials [81].

#### 6.2.3. SiPUU

**Figure 10:**
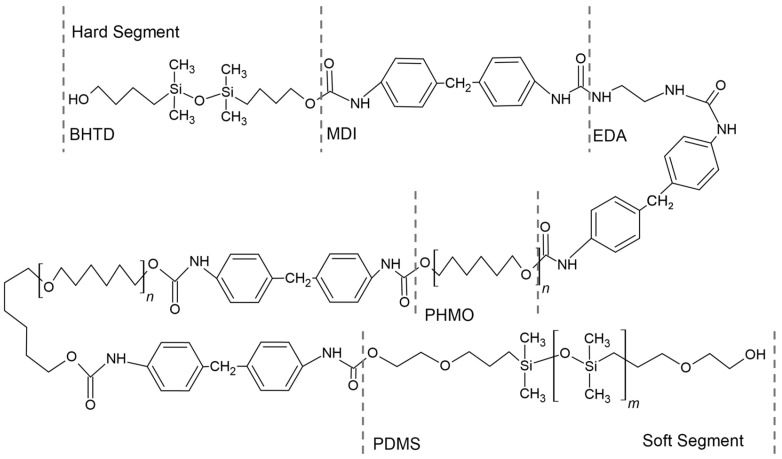
Chemical structure of SiPUU: soft segments are made of methylenediphenyl diisocyanate (MDI) linked with poly(hexamethylene oxide) (PHMO) and α,ω-bis(6-hydroxyethoxypropyl) polydimethylsiloxane; hard segments are composed of MDI and a mixture of 1,2-ethanediamine (EDA) and 1,3-bis(4-hydroxybutyl)-1,1,3,3-tetramethyldisiloxane (BHTD) in different molar ratios.

Although the current generation of siloxane polyurethanes (e.g., Elast-Eon™) possesses the necessary mechanical properties and biostability for different kinds of medical implants, biomedical applications such as heart valves require materials with improved mechanical features in addition to long-term biostability. SiPUU (Figure 10) is an improved biostable elastomeric polyurethane, composed of siloxane and polyurethane segments, often linked by urea groups. The siloxane segments provide flexibility, biocompatibility, and excellent resistance to thermal and oxidative degradation due to the silicon-oxygen backbone, the polyurethane segments improve mechanical strength and durability, and urea linkages enhance the polymer’s mechanical properties and thermal stability [82]. This polymer is obtained through a two-step synthetic process. The soft segments of SiPUU are made from a 20:80 (*w*/*w*) mixture of 4,4′-methylenediphenyl diisocyanate (MDI) linked with poly(hexamethylene oxide) and α,ω-bis(6-hydroxyethoxypropyl) polydimethylsiloxane: this combination has proven to be ideal for long-term biostability and good mechanical properties [83]. The hard segments are composed of MDI and a mixture of 1,2-ethanediamine (EDA) and 1,3-bis(4-hydroxybutyl)-1,1,3,3-tetramethyldisiloxane (BHTD) in varying molar ratios, and it has been shown how the tear resistance and mechanical strength increased by increasing the amount of EDA [84]. An in vitro degradation study showed that SiPUU is resistant to oxidation, even after undergoing chemical modifications, while retaining its good mechanical properties [85].

**Figure 11:**
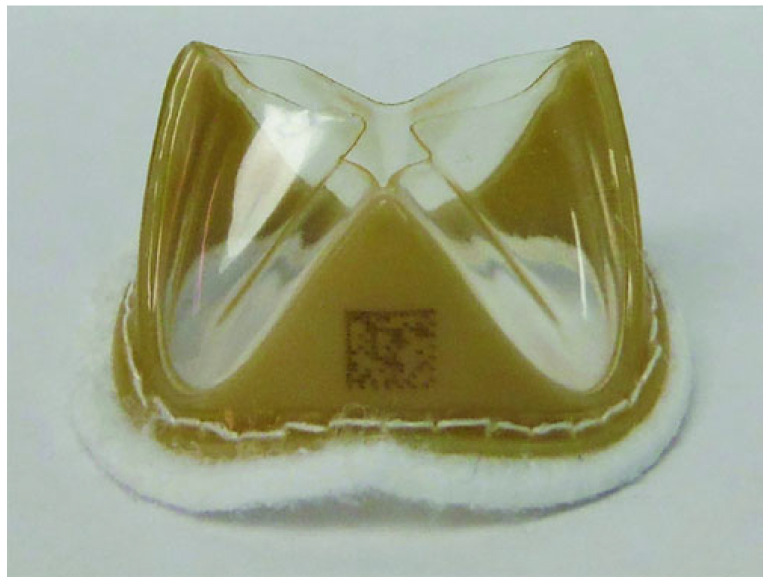
The TRIA valve for traditional open-heart surgery. Reproduced from [86], Wiley, 2020.

A SiPUU heart valve prototype was developed by Millson et al. [86]. The SiPUU (LifePolymer, LP, Foldax, Salt Lake City, UT, USA) valve, with the trade name of “TRIA Valve”, consists of a radio-visible polyetheretherketone (PEEK) stent, two or three specifically designed leaflets (depending on valve anatomical destination), and a PTFE felt sewing ring (Figure 11). TRIA valves were tested in accordance with the international standards, identifying no significant risk of toxicity or tissue injury [86]. Ex vivo thrombogenicity assay demonstrated nearly undetectable levels of platelet adhesion and fibrin deposition on polymer surfaces; moreover, TRIA valves showed exceptional hemocompatibility in two different large animal models. For sheep, eight sterilized TRIA valves were successfully implanted in the aortic position for 140 days: minimal inflammation adjacent to the aorta and the suture were observed, with no hemorrhagic evidence, thrombotic formation, or mineralization effect. A minimal amount of fibrous tissue coverage was demonstrated for the control leaflets, but no endothelialization was observed, while no significant health problems were detected for the sheep during the observation period [86]. These studies showed that the material exhibits ideal properties for polymeric heart valves.

In 2021, a study was published to provide a preliminary evaluation of the clinical effectiveness and safety of the TRIA aortic heart valve by Foldax Inc., the only company authorized by the FDA for this trial [87]. In total, 15 valves were implanted in 15 patients to assess clinical effectiveness endpoints (improvement in EOA [Effective Orifice Area]; improvement in NYHA classification, where the New York Heart Association (NYHA) Classification provides a simple way of classifying the extent of heart failure and classifies patients in one of four categories based on their limitations during physical activity; rate of adverse events; hemodynamic parameters) and primary safety endpoints (thromboembolic events, major hemorrhage, all-cause and valve-related death, other valve-related events like reoperation or explantation) within one year after implantation [87]. Between July 2019 and March 2020, 15 patients (14 men; mean age, 60.7 ± 7.2 years [range: 45–71 years]; mean body mass index, 32.9 ± 6.4 kg/m^2^ [range: 25.8–47.9 kg/m^2^]; mean Society of Thoracic Surgeons Predicted Risk of Mortality score, 1.39% ± 2.19% [range: 0.35–9.08%]) were enrolled. According to the study, anticoagulation with warfarin was initiated postoperatively. One patient was withdrawn intraoperatively due to valvular regurgitation. There were two deaths (on postoperative days 60 and 90) unrelated to the valve or procedure: one due to bleeding after elective surgery to remove a renal tumor, and the other due to hemodynamic collapse and cardiac arrest probably caused by pulmonary embolism after the discontinuation of warfarin in a subject with morbid obesity. Additionally, there was one lacunar stroke on postoperative day 172. Another patient experienced acute myocardial infarction caused by the thrombotic obstruction of the right coronary artery 92 days postoperatively. The TRIA LP heart valve showed significant improvements in transvalvular gradients (normalized pressure values from 33.3 to 9.5 mmHg), EOA values (from 1.2 up to 2.0 cm^2^), and NYHA functional class at one year follow up, with no patient remaining in functional class III or IV [86]. Safety outcome measures appeared comparable to those reported for bioprosthetic heart valves. On 16 April 2024, Foldax Inc. announced that “TRIA valves have surpassed 200 patient life years in human recipients. Patient life years represent the cumulative time the valve has been implanted in patients, offering insight into the duration of its use and its impact on cardiac function over time” [88].

**Figure 12:**
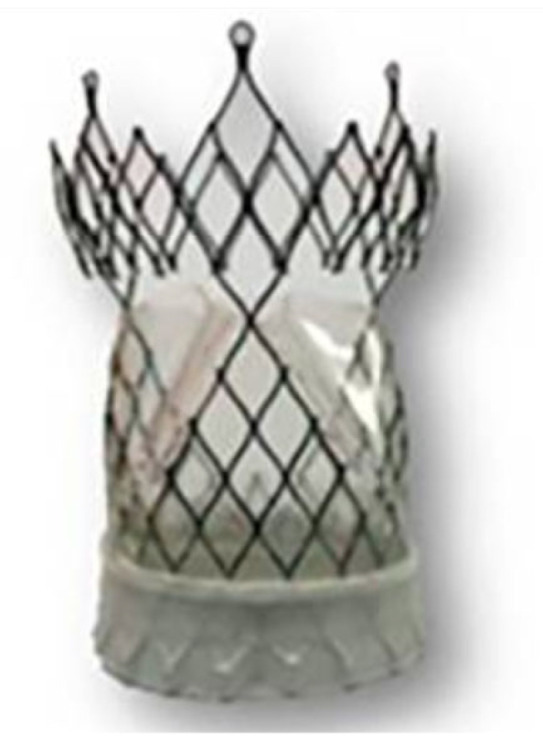
The TRIA valve for TAVI. Adapted from [90], MDPI, 2023.

Regarding transcatheter heart valves, in 2018, Steven J. Yakubov announced the development of a transcatheter TRIA heart valve manufactured by Foldax Inc. (Figure 12). The transcatheter TRIA is made of LifePolymer material, and its design has been optimized to increase durability and hemodynamics. The valve was investigated in vivo, being implanted in the aortic position of four animals (ovine models) with surgical annuloplasty rings (to simulate a calcified annulus). Two animals had suboptimal deliveries resulting in immediate explantation. In one animal, the device was placed too low in the annulus, resulting in migration to the left ventricle. The other two animals had successful deliveries and continued to 30 and 90 days: one of them had a small annulus (19 mm), resulting in mild acute paravalvular leak (resolved at 5 days) and mild central insufficiency due to incomplete device expansion. The mean gradient was 15 mmHg post-implant, 9 mmHg at 5 days, and 12 mmHg at 30 days with trace insufficiency. No valve thrombosis, leaflet tears, or tissue ingrowth was present. The sealing skirt was well integrated by fibrocellular tissue. The last animal was implanted with a 27 mm device in a 23 mm inner-diameter annular ring. The mean gradient was 6 mmHg immediately at 31-day follow up. Overall, the results showed good hemodynamics, the absence of calcification, thrombotic or fibrotic events after 30 days, and clean leaflets after 90 days [89].

#### 6.2.4. SIBS and xSIBS

**Figure 13:**
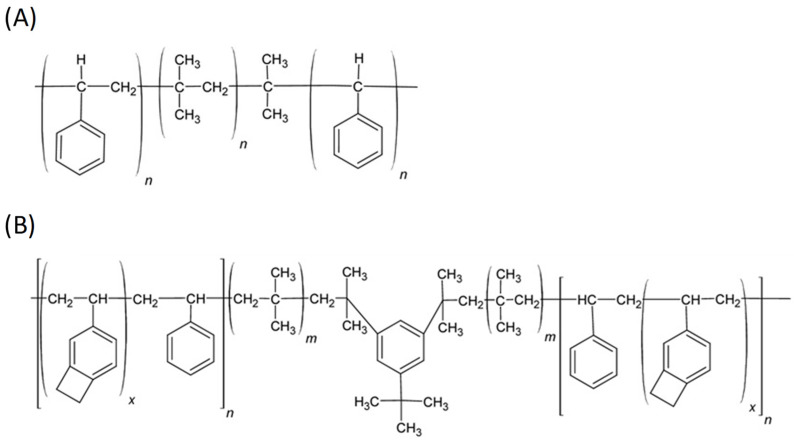
Chemical structures of SIBS (**A**) and xSIBS (**B**): SIBS is characterized by the presence of alternated polystyrene and polyisobutylene segments; xSIBS is made of poly (styrene-*b*-4-vinylbenzocyclobutene-*b*-isobutylene-*b*-styrene-*b*-4-vinylbenzocylcobutene).

Poly(styrene-block-isobutylene-block-styrene) (SIBS) (Figure 13A) is a thermoplastic block copolymer discovered in the early 1990s, with physical properties that overlap with those of silicon rubber and polyurethane. It remained unknown until the introduction of Boston Scientific Corporation’s (BSC) Drug Eluting TAXUS Coronary Stent in 2002 [91]. Due to its chemical composition, SIBS maintains oxidative, hydrolytic, and enzymatic stability throughout its lifespan in the body, making it biostable and associated with a relatively low foreign body reaction. SIBS is synthesized through the sequential polymerization of styrene and isobutylene monomers to form block copolymers, typically utilizing living anionic polymerization techniques. Its hardness can be varied by changing the amount of styrene [89]. However, the thermoplastic nature of SIBS results in poor creep properties, susceptibility to stress cracking, and fatigue failure [91,92]; it has poor gas permeability (which renders it more difficult to sterilize with ethylene oxide), and it is not sterilizable via gamma ray [91]. Thus, one of the critical aims of the researcher was to enhance the physical properties of the material, principally via fiber reinforcement. Gallocher et al. tried to reinforce SIBS with polypropylene, resulting in a significant improvement of mechanical properties, but unexpectedly high platelet deposition was observed on tissue samples [92]. The same results were observed with the reinforcement of SIBS with polyethylene terephthalate (PET, Dacron) fibers as performed by Wang et al. [93]: in this case, the valve failed in animal testing because of material failure and calcification. An improved version of this polymer, named xSIBS (Figure 13B), was developed by Innovia LLC (Miami, FL, USA), aiming to eliminate the significant dynamic creep of the thermoplastic SIBS that has limited its suitability for PHVs [94]. SIBS was modified to enhance its mechanical properties, resulting in a flexible, tear-resistant material with improved hydrodynamic performance, reduced thrombogenicity, and increased durability [95]. xSIBS is also an excellent candidate as a material for prosthetic transcatheter heart valves.

**Figure 14:**
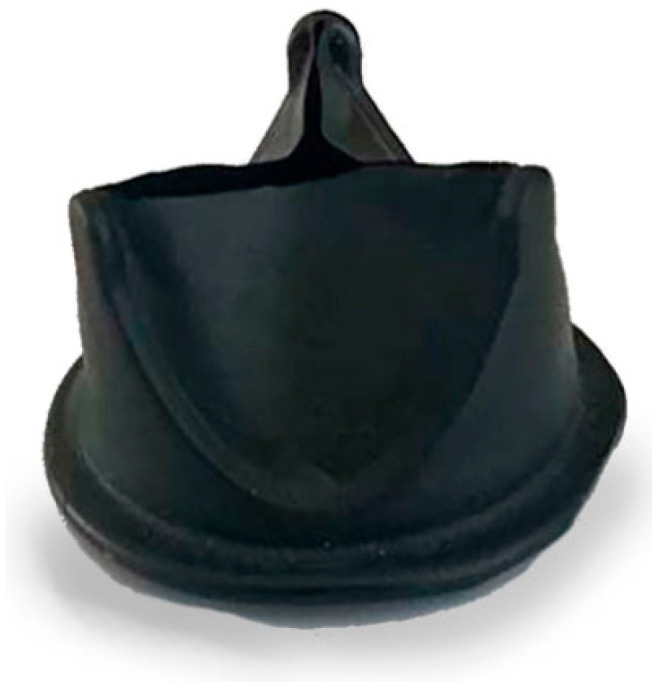
The Innovia valve (optimized design). Adapted from [90], MDPI, 2023.

The design of the SIBS valve underwent several improvements to ensure adequate hemodynamic performance and durability. First, Innovia LLC developed a polymeric trileaflet heart valve using SIBS. The valve design was changed several times, transitioning from a low to a medium profile. Moreover, the leaflet geometry was converted from a spherical to a cylindrical configuration, and the leaflet reinforcement was changed from low-density to high-density Dacron with the same nominal thickness. Furthermore, the leaflet fabrication process was enhanced from dip-coating to casting. These modifications have significantly improved valve durability by reducing the incidence of SIBS failure [94]. However, all these changes were not enough to receive approval for clinical use. In vivo studies on animals showed Dacron fatigue failure, calcification, and SIBS leaflet coating cracking. These issues were partially solved using xSIBS and an optimized design [94]. A novel xSIBS heart valve was developed by Claiborne et al. [94] with changes in the geometry. The leaflet shape was modified from cylindrical to hemispherical, and a flat leaflet profile was designed to maximize the coaptation surface. Additionally, to minimize the stress during the cardiac cycle, the radial cross-sectional profile of the leaflets was modified from uniform to variable thickness [94]. The optimization of the valve’s configuration and leaflet geometry was performed using parametric DTE methodology, which includes a series of simulations comparing the original Innovia valve, a benchmark biological valve, and the novel xSIBS valve. Enhanced performances were observed in the optimized valve in terms of durability and hemodynamic and thrombogenic resistance [94]. This optimized PHV was later called the “first-generation” Polynova surgical aortic valve (Figure 14).

SIBS and xSIBS have also gained traction in the development of TAVR. In 2009, Claiborne et al. developed a SIBS trileaflet heart valve mounted on a Nitinol self-expanding stent [96]. The leaflet thickness and optimal valve geometry were determined through a series of in vitro tests, proving to be capable of resisting migration and crushing forces exceeding those expected in vivo.

**Figure 15:**
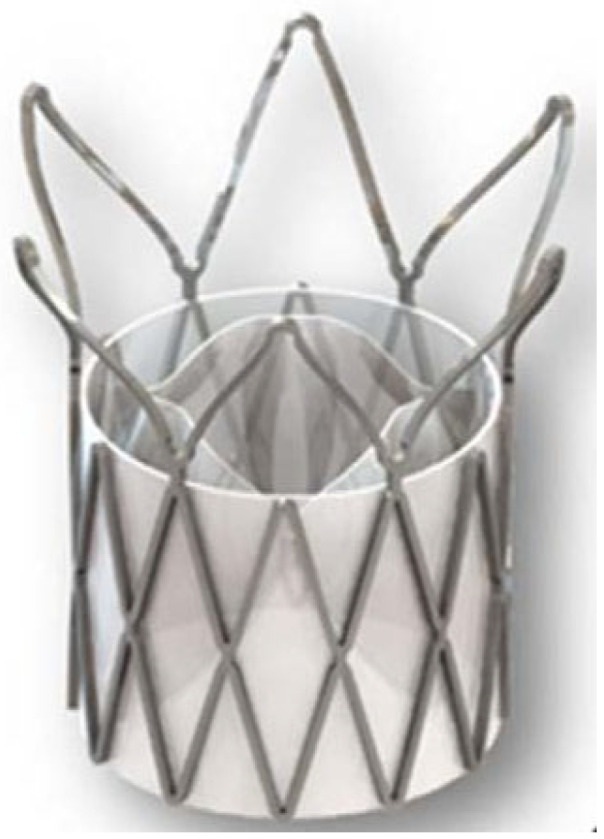
The Polynova valve for TAVI. Adapted from [100], Frontiers, 2023.

In 2018, Rotman et al. developed a novel polymeric aortic heart valve for TAVR made of xSIBS [97]. This new valve was derived from the SAVR valve previously described, with modifications regarding the fabrication process and enhancements in leaflet design, resulting in improved mechanical properties and durability. Rotman’s group proceeded with an in vitro study of the new valve [98]. The EOA remained stable with a gradual decrease in transvalvular pressure gradient and regurgitation. Additionally, calcification was significantly reduced, and no tears or surface damage were observed following crimping testing [98]. Despite better performances, the Polynova TAVR valve (Figure 15) exhibited the highest overall regurgitant fraction compared to the previous valves, indicating the need for further optimization.

In 2022, Kovarovic et al. introduced a novel “second-generation” polymeric TAVR valve [99]. The stent frame was redesigned to decrease the crimped volume compared to its predecessor, while also generating higher radial forces within the intended range of use to prevent crimping damage. Additionally, leaflets were optimized for larger-size devices, further reducing cyclic stresses. This second-generation polymeric TAVR valve is currently undergoing in vitro hydrodynamic testing and, subsequently, in vivo animal trials [99].

A novel advancement in the development of PHVs is the introduction of carbon nanofillers (CNTs) into SIBS [101]. In an initial study, CNTs were dispersed in chloroform using sonication following the addition of SIBS solution at different ratios. The optimal results were observed for SIBS films containing 4–6 wt% of CNT, which demonstrated higher tensile strength and conductivity compared to the original polymer, although with increased stiffness [101]. Subsequently, it was shown that using dodecylamine (DDA) to modify CNTs, characterized by a long non-polar alkane chain, significantly improved the dispersion of nanotubes in SIBS compared to unmodified CNTs [102]. As a result of this modification, the tensile strength increased and in vitro tests proved higher biocompatibility with no toxicity toward blood components, but high concentration of CNTs can lead to platelet aggregation, potentially causing thrombotic effects. Consequently, SIBS with higher molecular weight, reinforced with 1–2 wt% of modified CNTs, is one of the most promising materials for creating cardiovascular implants like heart valve prostheses [102].

#### 6.2.5. PVA and PVA-BC

**Figure 16:**
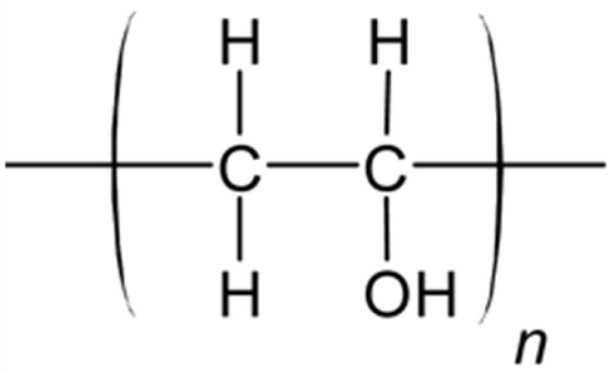
Chemical structure of poly(vinyl alcohol) (PVA).

Poly(vinyl alcohol) (PVA) is a hydrophilic polymer known for its advantageous properties, including excellent physico-mechanical characteristics, non-toxicity, biocompatibility, and high swelling capacity, making it highly suitable for various biomedical applications [103]. PVA is synthesized through the radical polymerization of vinyl acetate, resulting in poly(vinyl acetate) (PVAc), which is subsequently hydrolyzed to yield PVA (Figure 16). The polymer can be crosslinked either chemically or physically, but the physical crosslinking offers the advantage of eliminating residual amounts of toxic crosslinking agents compared to chemical methods [103]. Furthermore, by adding a filler (particles or fibers) into PVA and making PVA-based blends, desirable mechanical properties can be achieved. This enables the replication of different human tissue textures and properties, enhancing its potential for biomedical applications.

The exploitation of PVA for medical purposes originated between the 1950s and 1960s. However, its specific use in the biomedical field has become more prominent in recent decades. In the early 2000s, Wan et al. showed that the tensile properties of PVA hydrogel can be adjusted via freeze/thaw cycles to closely align with the physiological range of porcine aortic root [104]. They successfully developed a prototype of an expandable artificial heart valve stent using an injection molding technique with PVA hydrogel subjected to four freeze/thaw cycles [104]. In the same years, Jiang et al. developed a novel tricuspid valve completely made of PVA cryogel (PVA-C) [105]. In their study, they examined two different leaflet designs, resulting in two different sizes of the central orifice. The cylindrical stent was fabricated with PVA-C. The opening and closing phases of the valve prototype were successfully demonstrated on the bench by a cyclic flow tester. A unique advantage of the PVA-C heart valve is its ability to be temporarily compressed into a small ball, facilitating the insertion into the chest cavity through a small incision [105]. This feature is particularly suited for heart valve replacement via closed heart surgery, with the potential to reduce blood hemolysis and the incidence of bleeding complications, while ensuring an unobstructed central orifice during opening.

**Figure 17:**
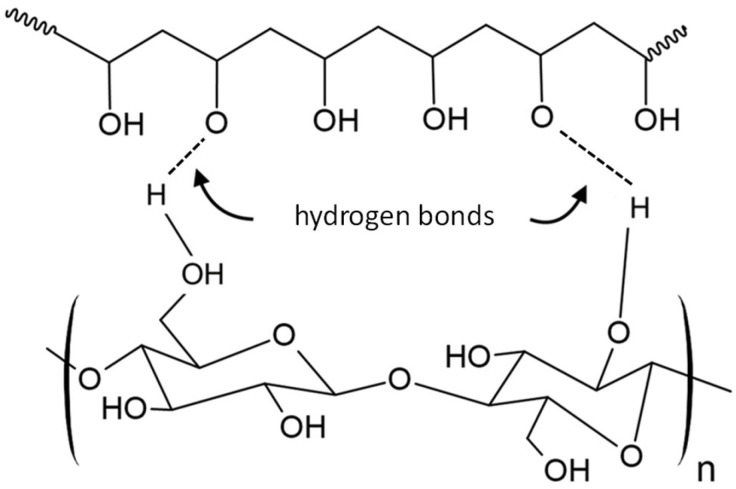
Chemical structure of PVA-BC: bacterial cellulose (BC) is linked to the poly(vinyl alcohol) (PVA) chain with hydrogen bonds.

In the following years, several PVA-based nanocomposites were developed and studied for biomedical applications, improving the mechanical properties. Wan et al. reinforced PVA with bacterial cellulose (BC) (Figure 17) [106], a nanomaterial with several valuable characteristics, including polyfunctionality, hydrophilicity, and biocompatibility [107]. They produced multiple samples of the PVA-BC nanocomposite with varying concentrations of PVA and BC. The incorporation resulted in a material with higher mechanical strength and greater stiffness compared to PVA alone, and it can be tailored to possess mechanical properties almost identical to those of cardiovascular tissues such as heart valve leaflets [108,109]. Despite these promising results, there are no current studies on the long-term in vitro and in vivo performances of this nanocomposite. However, other researchers have demonstrated the potential use of this material for the development of a TAVR prototype, but further discussion will be provided later.

Another material with promising properties for biomedical applications is the graphene oxide/polyvinyl alcohol (GO/PVA) composite [110]. GO/PVA composite hydrogel is commonly prepared using a freeze/thaw method. Compared to pure PVA hydrogel, it shows increased tensile strength, breaking elongation, and compressive strength. The incorporation of GO into PVA hydrogel does not affect the compatibility of PVA to osteoblast cells, and therefore, GO/PVA hydrogels should have many potential applications as biomaterials [110]. It is worth noting that among the methods for increasing the biocompatibility of blood-contacting devices, the surface can be altered by applying a hydrogel coating [111]. PVA can also be a promising polymer for long-term blood-contacting PHVs, considering issues such as fatigue resistance.

As mentioned before, PVA is also used for the development of transcatheter heart valves. Mohammadi et al. proposed a novel tricuspid percutaneous valve, with leaflets made entirely of PVA-BC nanocomposite [112]. The valve’s geometry was adjusted to prevent the sharp warping of the leaflets and eliminate the central opening orifice area when the valve is fully closed. It was combined with a Nitinol stent and a Dacron cover, and a specific cavity mold was designed for the fabrication of the valve [112]. Indeed, this model is considered for the mitral position, which currently lacks commercial implementation due to its significant technical challenges such as risk of paravalvular regurgitation, preventing migration to the left ventricle and providing a low profile with sufficient anchoring [113].

#### 6.2.6. LLDPE and HA-LLDPE

**Figure 18:**
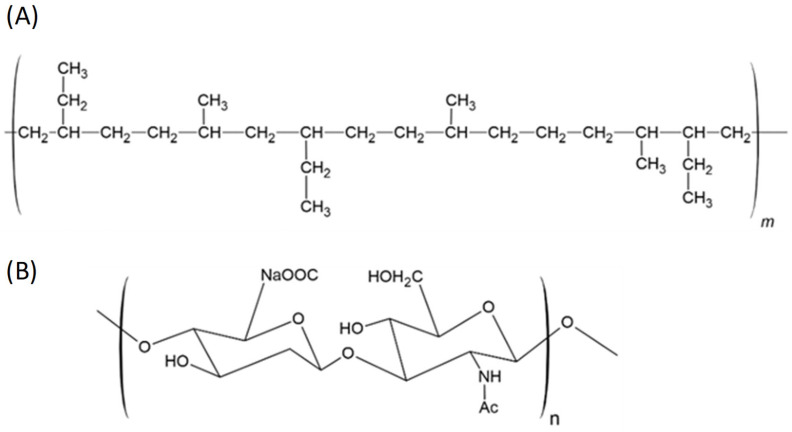
Chemical structures of linear low-density polyethylene (LLDPE) (**A**) and hyaluronic acid (HA) chain (**B**).

Linear low-density polyethylene (LLDPE) (Figure 18A) is a type of polyethylene (PE) with a linear polymer chain structure and a relatively low density compared to the other types of PE. LLDPE is synthesized through the copolymerization of ethylene with long-chain olefins. This structure provides the material with different properties, such as high tensile and tear resistance, low bending stiffness, and low shear stress sensitivity [90]. However, LLDPE does not provide sufficient biocompatibility for long-term blood interaction. Some studies have shown how hyaluronan (hyaluronic acid, HA), whose structure is shown in Figure 18B, incorporated into LLDPE can lead to improvements in the first polymer. HA is a naturally occurring polysaccharide found in tissue and body fluids of vertebrates and in some bacteria. Its structure contributes to the viscoelastic, hydrophilicity, and lubrication properties [114].

Prawel et al. evaluated the possible use of HA-LLDPE as a material for flexible heart valve leaflets [115]. HA-LLDPE materials were manufactured using the swelling method, a fast and simple technique that does not alter the mechanical properties of the LLDPE. The presence of HA reduced the static water contact angles for all the material samples [115]. HA-LLDPE was used to fabricate a trileaflet heart valve, which was assessed in a pulsatile flow loop system, demonstrating promising hemodynamic performances. Subsequently, the researchers evaluated the hemocompatibility of the material [116]. The surface was found to be cytocompatible, showing no significant cellular necrosis. Whole-blood clotting assay indicated that the HA-LLDPE surface significantly reduced clotting under static conditions due to a reduction in the intrinsic pathway activation and platelet adhesion/activation [116]. The study demonstrated the material’s potential for cardiovascular applications.

Yousefi et al. tested different LLDPE valve prototypes to find the best design able to improve the hemodynamic features [117]. The alteration of the aspect ratio between stent height and diameter, as well as leaflet arch length variation, led to the creation of nine different valve configurations. The researchers focused their study on configurations that ensure full commissure coaptation. For this purpose, they added short leaflet arches to valves with medium aspect ratios and omitted leaflet arches in valves with higher aspect ratios [117]. Consequently, six out of the nine configurations were analyzed. The study revealed that incorporating leaflet arches and adopting a higher profile offer several advantages. First, they result in a significant decrease in the RSS (Principal Reynolds Shear Stress), yielding better leaflet coaptation and reducing regurgitation percentage. However, it is important to note that a high stent profile might delay the reattachment of flow in the aorta and marginally increase the RSS. This increase could potentially lead to higher levels of hemolysis and blood damage. Furthermore, the introduction of leaflet arches leads to a substantial improvement in leaflet kinematics and heart valve hemodynamics by refining the low-profile design of the heart valve [117].

In recent years, HA-LLDPE has also played a role for transcatheter heart valves. A novel HA-LLDPE-based transcatheter heart valve, the HA-TAV, was developed and compared against two other commercially available TAVRs: the Medtronic Evolut and the Edwards SAPIEN 3 [118]. The hemodynamic parameters indicated that the HA-TAV surpasses basic requirements and stands on par with the leading prosthetic valves. Additionally, turbulent flow characterization in the HA-TAV demonstrates enhancements compared to the leading commercially available devices [118]. However, to evaluate the expected long-term durability of the novel valve, an accelerated fatigue test is necessary.

#### 6.2.7. SEPS and SEBS

**Figure 19:**
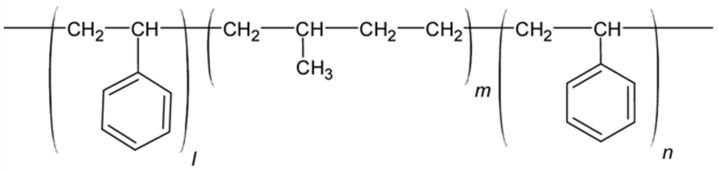
Chemical structure of poly(styrene-b-ethylene/propylene-b-styrene) (SEPS): the backbone is characterized by the alternation of polystyrene and polyethylene/propylene segments.

**Figure 20:**
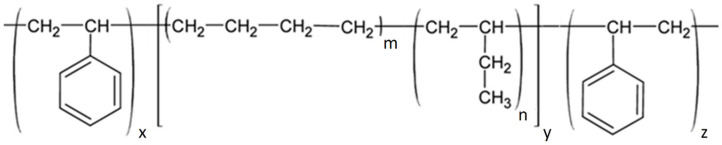
Chemical structure of styrene–ethylene–butylene–styrene (SEBS): the backbone is characterized by the alternation of polystyrene and polyethylene segments.

SEPS and SEBS are thermoplastic elastomers characterized by distinct chemical composition and versatile properties. SEPS, poly(styrene-b-ethylene/propylene-b-styrene) (Figure 19), is a styrenic block copolymer (SBC) with a hydrogenated isoprene unit as the mid-block [119]. It is a non-toxic material known for its thermal and oxidation stability, widely used in industrial applications and in medical tubing, films, and stoppers as well. However, its poor creep resistance and poor surface hydrophilicity lead to inferior biocompatibility when compared with other polymers [120]. SEBS, poly(styrene-b-ethylene/butylene-b-styrene) (Figure 20), is an SBC with a hydrogenated butadiene unit as the mid-block [121]. Despite its poor creep resistance on prolonged use, this material has good thermal, chemical, and UV resistance due to the presence of the saturated ethylene butylene unit [120]. With regard to calcification, a recent study indicated that a styrene block copolymer exhibits significantly lower susceptibility to calcification in vitro compared to a polyurethane valve [122].

**Figure 21:**
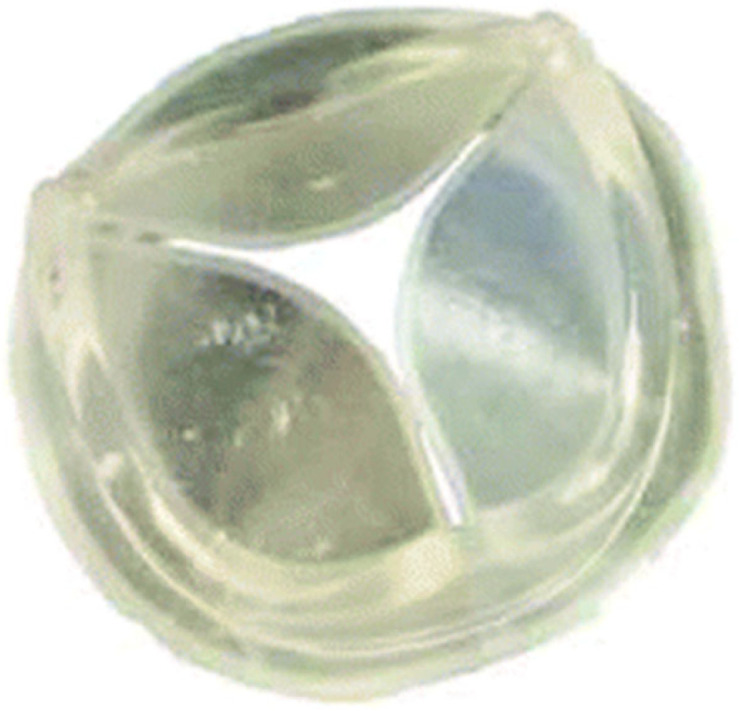
The Poly Valve. Adapted from [123], Royal Society of Chemistry, 2020.

Recently, Stasiak et al. developed and tested a novel polymeric heart valve, named Poli-Valve, manufactured by injection molding with SEPS and SEBS [123]. Both styrenic triblock copolymers were provided by Kraton Co. (Houston, TX, USA). Taking inspiration from the native valve, where collagen fibers are aligned to withstand mechanical stresses, the researchers used oriented styrene cylinders in the block copolymer to reinforce the polymeric leaflets. They performed in vitro and in vivo tests on various valve prototypes. The final design included a stiffer support structure made of SEBS29 and softer leaflets made of SEBS20, featuring cylindrically shaped leaflets with a concave leading-edge profile (Figure 21) [123]. The prototype’s performance exceeded the ISO standards in vitro, exhibiting excellent hydrodynamics and good durability equivalent to a 30-year life span. A short in vivo trial demonstrated excellent hemodynamics and biocompatibility, but further investigations are required [123].

#### 6.2.8. Other Nanocomposites

In recent decades, many nanocomposites have been created, studied, and tested for biomedical applications, with promising results. With specific regard to heart valves, some of these nanocomposites have shown excellent results when in contact with blood or subjected to high stress. In 2011, Volpato et al. used carboxyl-functionalized multi-walled carbon nanotubes (MWCNTs) as fillers in a polyamide 6 (PA 6) matrix to investigate the fabrication and the mechanical properties resulting from the addition of CNTs [124]. The PA6/MWCNT composite was produced by electrospinning, which allows for the dispersion and alignment of the MWCNTs within the polymer matrix, resulting in the formation of surface roughness. The researchers highlighted the encouraging results, noting that the fiber diameter, network morphology, and mechanical properties can be tuned to mimic specific target tissues [124].

Another promising nanocomposite is the poly(lactic-co-glycolic acid) (PLGA) enriched with carbon nanofibers (CNF) [125]. A study, with a 50:50 weight ratio of CNFs, showed that nanoscale roughness increases cardiomyocyte growth and enhances tensile strength and conductivity properties. Additionally, it was demonstrated that PLGA density can alter cardiomyocyte adhesion and proliferation, closely resembling native heart tissue [125].

Poly(propylene fumarate) (PPF), a linear aliphatic unsaturated polyester, has played an important role in biomedical applications after extensive investigation. With regard to cardiovascular applications, two studies have been reported: One study synthesized a novel segmented polyurethane (SPU) with PPF as the soft segments, resulting in a material with a regular structure, controllable deformation recovery ability, and tunable degradation rate [121]. The PPF-based polyurethane (PPFU) elastomer exhibited good mechanical properties, improved tensile strength, low cytotoxicity, and good biocompatibility, but even though further studies are needed before any clinical application [126]. The other study demonstrated that single-walled carbon nanotubes (SWNTs), particularly ultra-short SWNTs (US-tubes), are excellent reinforcing agents due to their superior mechanical properties [127]. This led to the development of a novel US-tube/PPF nanocomposite, which showed excellent biocompatibility and versatile surface chemistry, allowing the functionalization of the external surface with a variety of chemical moieties. Such modifications can enhance mechanical properties, hemocompatibility, and allow the in vivo monitoring of the degradation rate [127].

Graphene oxide (GO) is another component often used for the creation of nanocomposites. In 2012, Su-xing et al. developed a range of functional GO based on the biomimetic monomer MPC (GO-g-pMPC) and then prepared polyurethane (PU)/functional GO nanocomposite films (PU/GO-g-pMPC) [128]. Mechanical results and TEM analysis confirmed the successful synthesis. Additionally, the material demonstrated improved resistance to protein adsorption and platelet adhesion, indicating good hemocompatibility [128]. The following year, Jin et al. synthesized a novel exfoliated polyethylene/modified GO nanocomposite (PE/GO-MPC) via melt intercalation. At 0.2 wt% GO-MPC content, there was a significant increase in tensile strength and elongation at break. The material also exhibited excellent hemocompatibility and antimicrobial activity [129]. Both nanocomposites require further study, but they are expected to eventually be used in biomedical applications.

## 7. Manufacturing Techniques

For the production of a heart valve, manufacturing techniques can greatly vary depending on the desired mechanical properties or the specific 3D shapes needed for valve leaflets and geometrical configuration. Numerous chemical and mechanical processing steps can be required, but some common techniques for polymers can be considered. The choice of the most convenient technique largely depends on the type of polymer: for instance, thermoplastics can be processed using heat or solvents (if they are sufficiently soluble), while thermosetting materials require molding techniques. A brief description of the most widely used manufacturing processes for PHVs follows.

### 7.1. Injection Molding

**Figure 22:**
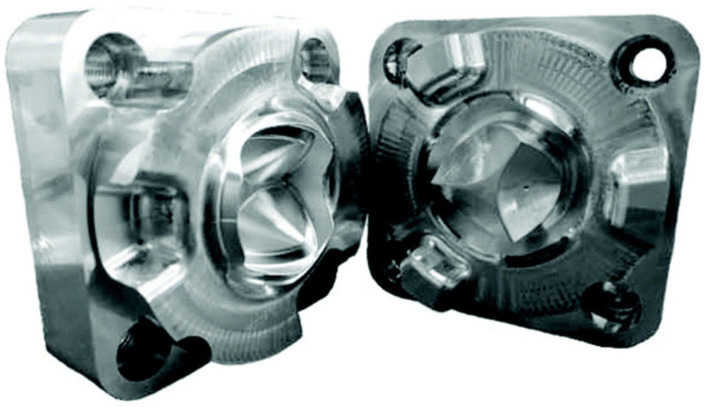
Aluminum inserts for the injection molding of the Poly Valve. Adapted from [123], Royal Society of Chemistry, 2020.

Injection molding is a widely used manufacturing method for creating PHVs due to its precision and efficiency in forming complex structures [130]. The process begins with the preparation of the polymer, typically in granules, which is heated up to a fluid state. The melted polymer is then injected under high pressure into a mold cavity designed to exactly reproduce the shape and dimension of the desired heart valve (Figure 22). The polymer is packed tightly into the mold to ensure it fills all the details of the cavity. Once injected, the mold and the polymer are cooled; thereafter, the mold is opened, and the newly formed object is carefully removed [123]. The resulting product may undergo further processes to remove any imperfection or excess materials. This method offers relatively inexpensive and highly reproducible manufacture.

Stasiak et al. manufactured the novel Poli-valve PHV through the injection molding of styrenic triblock copolymers with a cylindrical microstructure [123]. The resulting valve exhibited excellent mechanical properties, hemocompatibility, and promising results in short-term in vivo tests [123]. Alternatively, Sacristàn et al. opted for injection molding the valves in four-piece molds to reduce production costs, which resulted in variability in the leaflet thickness [131]. Later, it was shown that this valve had limited hemodynamic performance [132]. With a multistep approach, different polymers are introduced into the mold at different times so that multi-material structures can be developed [130]. Weber et al. proposed a novel multiple-step injection molding process to produce multicomponent heart valves made of fibrin and elastin-like recombinant gels [133]. The researchers demonstrated the potential to imitate the structure of native heart valves by incorporating different scaffolds, cell types, and components for both the leaflets and wall.

### 7.2. Compression Molding

Compression molding is a manufacturing process primarily used for forming thermosetting plastics and some thermoplastics. A pre-measured amount of polymer is placed into an open, heated mold cavity; the mold is then closed, and heat and pressure are applied, causing the material to flow and conform to the shape of the cavity [134]. This technique enables the manufacture of leaflets with controlled thickness and the desired mechanical and physical properties. However, for medical applications, improving the surface quality of additively manufactured parts after the building process is still critical because, for example, in heart valves, surface topography can influence thrombus development. Early silicone valves were formed in one piece by heating the mold up to 180 °C under high pressure and curing at 200 °C for 4 h [48]. These valves exhibited satisfactory short-term in vivo functionality [135]. Later, Roe et al. addressed some issues by casting thermocompression molds with different materials for the leaflets and sewing ring [51], achieving better results in clinical applications, with four patients surviving several months [50]. In 2013, Claiborne et al. developed a novel trileaflet polymeric heart valve made of xSIBS using a custom compression molding process [96]. More recently, Masheane et al. developed a novel compression molding process for PHVs, using additive manufacturing technologies to produce white resin molds [134].

### 7.3. Dip Molding

Dip molding is a manufacturing process, also known as dip coating, used to create thin-walled, hollow parts by dipping a mold or mandrel into a polymeric solution. This process is simple and cost-effective; it is suitable for producing items with uniform wall thickness and smooth surfaces. First, a mold or mandrel with, for example, the desired leaflet geometry is immersed into the polymeric solution, typically heated to a temperature ranging from 60 to 80 °C [136]. After removal, a layer of solution adheres to the mold. Thereafter, the solvent is removed by heating, and a thin solid polymeric film is formed on the mold. These steps can be repeated to achieve the desired polymer thickness. The valve frame (or stent) can be dipped together with the mold initially or in subsequent steps, facilitating a seamless transition and strong bond between the leaflets and the frame, even when using different materials [136].

Since the 1990s, this process has been used for manufacturing PHVs. In 1992, Jensen et al. produced a novel polyurethane trileaflet heart valve by dip coating, achieving optimal results during in vitro tests in terms of durability and biocompatibility, and also encouraging results in animal tests [137]. In 1996, in order to develop a novel polyurethane heart valve, Mackay et al. carried out a well-controlled dip process and formed valve leaflets integrated with their supporting frame in a single step, producing a valve comparable to the bioprosthetic ones [71]. In the early 2000s, Daebritz et al. used a dropping technique to deposit PCU onto the mold in a controlled way, enabling variation in the thickness across the leaflets. Then, they coated the entire valve, including the stent made of a harder PCU, ensuring a durable bond between the leaflets and the stent [74,118,138]. More recently, Rahmani et al. realized a semi-stented aortic valve prototype with a novel automatic dip coating process to improve reproducibility [78]. Furthermore, the researchers used the automatic enhanced manufacturing process based on dip coating controlled by robots to develop a novel TRISKELE valve [80].

### 7.4. Melt Blending

Melt blending is a process used to mix different polymers or polymeric composites in their liquid state to achieve homogeneous material with enhanced properties. It is often used as a preliminary step before other processing techniques such as injection or compression molding.

A composite is any material whose structure consists of two or more macroscopically identifiable components working together to attain better properties [139]. Composites are increasingly used for heart valves. Melt blending is the preferred method for preparing polymer nanocomposites with a thermoplastic or elastomeric polymeric matrix. The process begins with the selection of base polymers and any additional material or filler, for example, nanoparticles, that are intended to enhance specific properties like mechanical strength, flexibility, or biocompatibility. The selected materials are heated to their melting point in an extruder (or in a similar device). Once melted, they are mixed thoroughly to ensure a uniform distribution of all components. The mixing can be controlled to achieve the desired characteristics such as viscosity and homogeneity. After mixing, the melted blend is cooled and solidified through a different type of molding process, like injection or compression molding [140]. The main advantage of this method is that production rates and throughputs can be high. Since it does not require organic solvents, melt blending generates less chemical waste compared to other processes [141]. Despite the increasing use of composites and nanocomposites for the production of PHVs, many natural polymers cannot be melt-processed: they may degrade before or upon melting, or they may contain components that are unable to withstand high-temperature processing, such as proteins or drugs [141].

### 7.5. Additive Manufacturing

Additive manufacturing (AM), also known as 3D printing, is an advanced technique used to produce a wide range of objects. For biomedical applications, AM allows the production of complex devices and implants, including patient-specific items. Regarding PHVs, AM builds the structure layer by layer from a digital model, creating intricate designs that closely mimic the natural anatomy of heart valves. The layer-by-layer construction method facilitates the incorporation of complex geometry and specific material properties, essential for the functional and mechanical performance required for heart valve applications.

**Figure 23:**
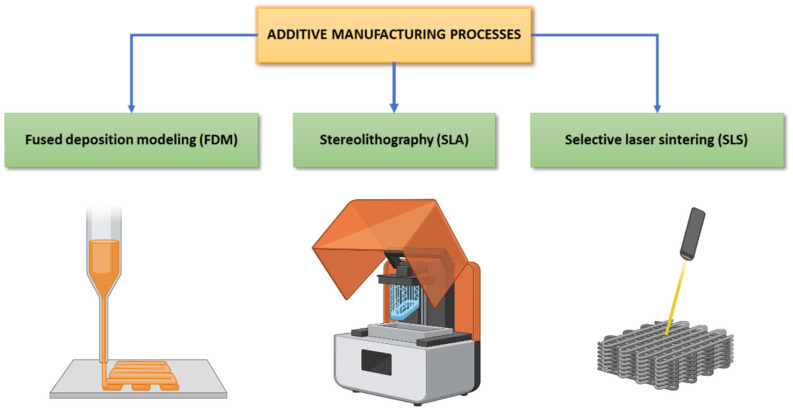
Examples of additive manufacturing process. Created in BioRender. Bagno, A. (2025) https://BioRender.com/k95b608.

AM includes different techniques that can be used depending on the desired product and its intended properties. The three main types are the following (Figure 23):

- Fused deposition modeling (FDM): It is an AM extrusion process that involves feeding a filament feedstock (polymer blend) through an electric motor-controlled pinch roller into a heated extrusion head. The filament melts as it moves through the head and is extruded through a nozzle [142]. The print head’s planar x-y motion, combined with the z motion of the building stage, allows the layer-by-layer deposition of the fused material. A second nozzle can deposit temporary supporting material. This technique is inexpensive, fast, and relatively simple, and can be expanded to multi-material printing using multiple nozzles [142]. However, it is limited to thermoplastic polymers with favorable viscosity, and due to the heated process, cells or temperature-sensitive biological components cannot be incorporated [141]. The final printed items can exhibit a layer-by-layer effect, particularly on sloping surfaces, which may reduce surface smoothness and require further refinement steps.
- Stereolithography (SLA): The manufacture of 3D objects by SLA relies on the spatially controlled solidification of a liquid resin through photopolymerization [143]. A digital light projector with a computer-driven building stage or a computer-controlled laser beam illuminates a pattern on the resin’s surface. The resin solidifies to a defined depth, determined by the energy of the UV beam/light, making it adhere to the support platform. After the photopolymerization of the first layer, the platform is moved away from the surface, and the newly exposed layer is coated with liquid resin. A pattern is then cured in this second layer. Since the curing depth slightly exceeds the platform step height, it ensures good adherence to the previous layer [143]. These steps are repeated to construct a solid, three-dimensional object. After excess resin is drained and washed off, the desired structure is obtained. Compared to other AM techniques, SLA has the highest print quality in terms of complexity and accuracy [141]. Complex internal features and cell viability can be achieved. However, the main limitations of this process are due to the limited availability and the high cost of photopolymerizable materials.
- Selective laser sintering (SLS): With this technique, a machine uses fine powder material to fabricate a 3D object based on a digital model. The powder is selectively heated with a laser beam to a point where the surface tension of individual particles is overcome, causing them to fuse together, solidifying layer by layer [144]. After each layer is finished, the supporting bed is lowered, a new layer of powder is spread with a roller, and the sintering process is repeated. Once the object fabrication is completed, the excess powder is removed and recycled [144]. SLS offers high accuracy and material versatility, but fabricated parts often require post-processing to improve surface quality. Polymers in fabricated parts may shrink or warp due to thermal distortion, and bioactive molecules cannot be incorporated due to the high temperature used [141].

AM technologies have the potential to revolutionize the manufacture of heart valves by enabling the creation of personalized, complex constructs. Improving printing resolutions, expanding the range of printable materials, and integrating multiple 3D printing technologies with other manufacturing techniques will pave the way toward the production of clinically usable and durable heart valves [145]. Although the majority of applications and studies of these processes have been concentrated in the field of tissue-engineered heart valves (TEHVs), recent attention has also focused on producing new valve prototypes or individual components of PHVs. In 2021, Cavallo et al. proposed a novel polyurethane-based self-expandable trileaflet aortic valve, with the stent printed with a fused deposition modeling (FDM) 3D printer: promising results in terms of valve crimping capabilities, durability, fluid dynamic performance, and hemocompatibility were obtained [146]. In 2023, a novel 3D printable heart valve was proposed by Schröter et al. By modifying the lateral profile of the triangular leaflets and 3D printing in silicon, they obtained a prototype that exhibited overall characteristics comparable to those of both currently adopted biological and mechanical prostheses [147].

### 7.6. Solvent Casting

Solvent casting is a widely used technique in biomedical applications due to its simplicity and effectiveness in creating thin films, complex structures, and membranes. This process involves several steps [148]. First, the desired polymer is dissolved in an appropriate solvent to create a homogeneous polymer solution [141]. The polymer solution is then poured into a mold that defines the shape of the desired object. The polymer is left to dry by solvent evaporation so that the solvent evaporates and the polymer precipitates as a solid film. The thickness and uniformity of the film can be controlled by adjusting the concentration of the polymer solution and evaporation conditions [148]. Once solidified, the polymer is carefully removed from the mold. Post-processing steps, such as annealing or cross-linking, may be applied to enhance the mechanical properties and stability of the material. This process has played a role in manufacturing various PHVs over the years. For instance, the Angioflex heart valve, made of polyurethane, showed excellent results in calcification tests compared to other prosthetic heart valves [149]. For SIBS valves, a study demonstrated that solvent casting with a 25 µm SIBS coating thickness provides optimal biocompatibility, as evidenced by in vitro and in vivo tests [150]. More recently, SIBS has been studied to improve its applicability in PHVs. The material, dissolved in chloroform, exhibited the ability to replicate native leaflet functions with minimal risk of perforations, leaflet stress accumulation, ruptures, and tears [151].

## 8. Overall Consideration of Valve Design and Geometry

The choice of the design is a crucial step for the development of artificial heart valves. Unlike bioprosthetic valves, PHVs can be virtually designed in any shape, which emphasizes the importance of defining structural design strategies. In particular, the geometry of the leaflets significantly influences the performance and durability of the valve. Several studies demonstrated that a geometry designed to mimic physiological flow patterns improves the hemodynamics and reduces the risk of thrombosis [152], while abnormal flow patterns can increase stresses on the arterial walls, leading to dilatation and potential aneurysm development. Furthermore, leaflet geometry can also impact the rate of calcification [73].

There are several key requirements in heart valve design:

1. The valve must fit well within the recipient’s anatomy.
2. The leaflets should offer minimal resistance to forward flow and rapidly open at a minimal variation in systolic transvalvular pressure.
3. They must ensure adequate sealing in the closed position to prevent/minimize the backward flow.
4. Efforts should be made to reduce damage to blood cells and thrombogenic effects.
5. Stress peaks in the valve components should be kept as low as possible throughout the cardiac cycle to ensure durability [153]

A geometry that closely matches the natural valve configuration is desirable. Various designs have been proposed throughout the development of PHVs, but none can perfectly match the complexity of a natural valve.

Until the early 2000s, valve designs substantially relied on experimental methods to assess their performance, often based on relatively straightforward mathematical equations that describe the geometry. In recent years, computational methods have significantly advanced in predicting the performance of any given valve design. Numerical simulations, such as structural finite element analysis (FEA), computational fluid dynamics (CFD), and the integration of fluid–structure interaction (FSI) physics, have become pivotal in optimizing valve designs and enhancing performance [136]. FSI models, the latest computational tool, combine solid structural physics with fluid flow physics, encompassing their highly nonlinear interaction. This distinguishes them from traditional computational fluid dynamics simulations, which typically assume fixed solid structures, and conventional structural finite element simulations, which often simplify the fluid effects to a fixed pressure distribution.

With regard to PHVs, over the years, both bileaflet and trileaflet valves have been studied and developed, but the majority of the investigations have focused on the trileaflet design. This is because trileaflet configurations are geometrically similar to natural pulmonary and aortic valves, which are the most frequently diseased valves due to their workload.

### Valve Design over the Years

The bileaflet mitral valve was one of the first artificial heart valves developed by Braunwald et al. in 1959 [67]. The valve was made of Dacron fabric enriched with PU in a mold, and its shape was obtained from casts of human and animal valves. Regarding bileaflet valves, a more recent study proposed a novel polymeric valve made of PCU [154]. This valve features an oval stent with two asymmetric struts supporting a small posterior leaflet and a large anterior one, mimicking the natural mitral valve morphology. It features a flat leaflet configuration and variable leaflet thicknesses (between 100 and 300 µm), encouraging physiological flow patterns [154]. In vitro and in vivo tests showed promising results, such as no thrombi formation, negligible regurgitation, and no signs of structural damage or calcification. However, the valve never reached the clinical phase.

Many designs of trileaflet valves have been studied over the years:

1. In 1958, Roe and Moore proposed cone-shaped leaflets, showing minimal resistance to systolic flow and promising hemodynamics and physiological efficacy [48,135].
2. In 1977, Ghista and Reul proposed an optimized design for achieving smooth washout, good coaptation, and minimal leaflet stress [155]. After the analytical study defined by Chong, based on the subtending angles and principal radii of curvature [156], the optimized design facilitated tangential flow and mutual support of the leaflets along the entire closure rim, achieving durability in vitro of more than 350 million cycles [155].
3. In 1982, Wisman et al. proposed a trileaflet PHV made of segmented polyurethane [157]: the valve was composed of hemicylindrical leaflets and a flexible support, and it showed good efficiency in vitro, but dystrophic calcification and thrombosis arose in animal tests [157]. Later, other studies were proposed, such as one on valve geometry using a cylindrical model, which showed important considerations regarding reduced dilatation pressure and low bending strains [158].
4. In 1994, Lean and Fisher proposed a novel design for a polyurethane heart valve [159]. They started by analyzing previous leaflet geometries and demonstrated that increasing the radius of curvature at the base of the leaflets enhanced opening characteristics. A new geometry, termed ‘alpharabola’, was proposed, which has a variable radius of curvature and a varied leaflet thickness, leading to a reduced opening pressure by over 40% and a minimal pressure differential [159].
5. In 1995, Mackay et al. developed a novel polyurethane valve that comprised a cylindrical support frame and, in the closed position, a leaflet shape defined by different conic sections: hyperbolic in the circumferential and elliptical in the radial direction [71]. Furthermore, hyperbolic parameters were adjusted to allow efficient coaptation with minimal stretching of the leaflets. The valve showed good hydrodynamic performance and durability without any sign of failure [71].
6. In 2001, the same researchers proposed a novel design for a trileaflet heart valve with conical geometry for the leaflet region next to the frame and a spherical upper leaflet region, allowing facilitated opening and closing phases, good coaptation, and a stable closed position [160]. The valve showed improved hydrodynamic function, with a greater EOA and reduced regurgitation, and the pulsatile flow results showed a lower total energy loss.
7. In 2003, Jiang et al., during the development of a heart valve made of PVA, proposed two novel designs based on the hyperbolic geometries described by Mackay’s group, defining leaflet shape using a hyperboloid of revolution around an axis and an arc subtending two straight lines [105]. The second one provided better control of the central opening and leaflet curvature, but both designs experienced gaps between adjacent leaflets and a large central orifice when the valve closes [105].

After the early 2000s, researchers started using more efficient computational tools to develop better designs for heart valves:

1. In 2008, Mohammadi et al. developed a novel PVA-BC heart valve, and by using Bezier surfaces (a spline that could be easily altered by manipulating control points in CAD software) combined with structural FEA and including material properties (i.e., the elastic moduli in orthogonal directions), they were able to analyze and select the optimal geometry, addressing the shortcomings of previous hyperboloid designs [106]. Even though fluid flow was not accounted for in the analysis, structural simulations demonstrated favorable opening and closing dynamics and stress distributions within the physiological range [106].
2. In 2010, Burriesci et al. proposed a novel polymeric heart valve design optimized by means of FEA [161]. The geometry, specifically conceived to minimize stress amplitudes in the leaflet and maximize the geometric orifice area, was defined as a ruled surface between the intersection of the stent cylinder with a plane and an arc joining the commissures. In vitro tests proved a larger EOA compared to Mackay’s ellipto-hyperbolic geometry, as well as reductions in regurgitation and transvalvular pressure drop [161]. Different studies have proven that a larger EOA, like that exhibited by valves with the thinnest leaflets, is preferable due to lower pressure differential, regurgitation, and energy loss [78]. This design was later used by Rahmani et al. in developing the novel TRISKELE transcatheter aortic valve [78].
3. Li and Sun investigated around 500 distinct leaflet designs by means of simulations of TAV closure under nominal circular deployment and physiological loading conditions. This study was aimed to observe how variations in leaflet geometry can affect valve peak stresses in both circular and elliptical configurations [162]. The optimization study led to the identification of an optimal leaflet design capable of reducing peak stress on the TAV leaflets by approximately 5% compared to the original design.
4. In 2015, Gharaie and Morsi utilized an FSI model to optimize and evaluate the performance of their design [163]: they compared previously published data with predicted results to validate the FSI model, developing a design characterized by two curves (radial and circumferential). These curves were optimized to achieve reduced stress concentration in the leaflets and minimal regurgitation. The optimized valve prototype demonstrated high hemodynamic performance with the absence of damage due to the stress concentration during the entire cardiac cycle [163].
5. In 2018, Ghosh et al. developed a polymeric SAVR valve using the FSI technique with a boundary-fitted method, the Arbitrary Lagrangian–Eulerian (ALE) method, which deforms the fluid cells according to the structural motion [164]. The valve showed a large opening area and a high flow rate, while the wall shear stress distribution and mechanical stress magnitudes were stable, demonstrating the enhanced performance of the prototype.
6. In 2021, Farokhi et al. developed a double-coupled fluid–structure interaction model using ALE and steered adaptive mesh (SAM) to investigate the impact of valve elasticity and valve positions on hemodynamics and solid parameters [165]. The simulation results indicated that a lower elastic modulus causes an increase in the EOA. Furthermore, the results of valve positioning showed that, when the valve is closer to the sinuses, greater EOA and lower stresses imposed on the leaflet are achievable [165].
7. Zhou et al. studied the correlation between valve thickness and material properties [166]. They utilized the FSI analysis to obtain a more accurate solution of the stress and strain distribution, EOA, and the regurgitation fraction of the valves with varying thicknesses across three different materials: Carbothane PC-3585A, xSIBS, and SIBS-CNTs. For each valve, they found specific optimal constraints for thickness based on the elastic modulus of each material: a smaller elastic modulus like the one of Carbothane PC-3585A allowed the production of a thicker valve (>0.3 mm); conversely, with an elastic modulus greater than that of xSIBS (2.8 MPa), excellent results can be obtained with a thickness less than 0.2 mm [166].

A summary of the evolution of polymeric heart valves over the last decades is illustrated in Table 2. Materials used for prosthetic heart valves production, processing methods, and general characteristics are depicted in Table 3.

## 9. Potential Complications Associated with the Use of PHVs

Before any clinical translation of PHVs, it is crucial to consider potential complications observed in vitro and in vivo studies over the years. Despite advances in valve design and surgical techniques, the replacement of a diseased valve with a prosthetic one is not yet a definitive cure for patients. The native disease is often supplanted by prosthetic valve disease, with clinical outcomes influenced by prosthetic valve hemodynamics, durability, complications related to the device, and patient response. Many of these complications can be prevented, or their impact minimized, with careful medical management, the periodic monitoring of valve functionality, or, in the case of PHVs, through modifications of material structure, surface properties, or overall valve design. For PHVs, major potential complications are associated with the following aspects:

1. Hemocompatibility: Generally speaking, it is one of the major factors limiting the clinical applicability of all blood-contacting biomaterials. Adverse interactions between material and blood must be extensively analyzed to prevent the activation and/or destruction of blood components. The protein layer initially adsorbed on the biomaterial surface primarily triggers adverse reactions, such as coagulation activation via the intrinsic pathway, leukocyte activation leading to inflammation, and platelet adhesion and activation, which can result in decreased blood cells and/or thrombus formation [167]. Prior to clinical application, the hemocompatibility of blood-contacting materials has to be investigated, as outlined in ISO 10993-4 [168]. This standard describes five categories for analysis: thrombosis, coagulation, platelets, hematology, and immunology.
2. Thrombosis: It is an in vivo phenomenon that results in the partial or complete occlusion of a blood vessel or device due to blood clot formation (coagulation). Events such as intraoperative stroke, transient ischemic attacks (TIAs), sudden death, and myocardial infarction may be due to prosthesis-generated thromboemboli [169]. Proteins’ adsorption has a crucial effect in determining the level of thrombogenicity of any given material. Unlike mechanical valves, PHVs significantly reduce thrombotic risks and, as a consequence, the amount of anticoagulation required [100]. This reduction is important because anticoagulation often results in bleeding episodes, which can cause death, stroke, reoperation, or hospitalization. Indeed, MHV recipients require lifelong anticoagulation, typically with vitamin K antagonists (e.g., warfarin), which brings significant challenges, including frequent blood analyses, possible drug–drug interactions, dietary and activity restrictions, medication costs, and the need to travel long distances for anticoagulation monitoring [170].
3. Calcification: It involves the gradual accumulation of calcium salts over and inside valvular tissue, potentially leading to various complications. While PHVs are designed to be less prone to calcification than bioprosthetic ones, they are not entirely exempt, prompting numerous studies aimed at minimizing or eliminating this event. Calcific deposits commonly occur in the commissural and basal regions of the cusp. This phenomenon can lead to adverse outcomes in both preclinical studies and long-term implantation. Growing calcific masses can block the leaflets, causing structural valve deterioration and potentially resulting in stenosis or regurgitation [171]. PHVs have a distinct advantage over traditional bioprostheses because they lack animal-derived proteins, such as Neu5Gc and αGal: this may lead to longer durability [100].
4. Infection: Infection of the valve and/or surrounding tissues (prosthetic valve endocarditis) is a severe complication that may necessitate antibiotic treatment and eventually valve replacement [172]. The risk of endocarditis associated with PHVs has not been extensively studied, necessitating further research to clearly define these risks compared to BHVs and MHVs. Further complications related to infective endocarditis include congestive heart failure, embolization, and paravalvular abscesses.
5. Degradation: Polymers must withstand the aggressive assault due to the chemically severe and mechanically dynamic physiological environment [173]. Biostability is the term used to identify the ability to not experience any measurable deterioration over the course of service: it has to be thoroughly tested by exposing the polymer in vitro and/or in vivo, but the duration of exposure is usually a fraction of the intended exposure time of the device. In-service degradation is addressed in ISO 10993 (Biological Evaluation of Medical Devices Part 13 (polymers)). In particular, Section 4.1.4 from ISO 10993-13 [174] (allowing for accelerated testing) is not recognized by the FDA: the current FDA standard requires a six-month preclinical exposure in combination with an oxidation challenge, or alternatively, a two-year preclinical exposure, where the polymer must perform comparably to (or better than) the historical materials [173]. Degradation is due to chemical reactions whose byproducts can result in adverse effects inside the body (affecting its biocompatibility), even changing the intended performances of the device. Typically, polymers are exposed to hydrolysis and oxidation, which are related to the chemical structure and morphology of the polymer [90].
6. Mechanical complications: Some complications related to PHVs can be categorized as mechanical issues. Due to the repetitive opening and closing cycles, PHVs are susceptible to fatigue failure that, over time, can lead to the development of cracks and eventually structural failure. As previously mentioned, developing proper valve geometry is a crucial step, along with conducting appropriate fatigue tests, often under conditions more severe than those for a native valve. Additionally, the cyclic application of mechanical stress can cause wear and tear, leading to the thinning of valve leaflets and potential rupture. This scenario depends on the given material or, once again, on the leaflet’s configuration.
7. Failure mode: While the mechanism of tissue degeneration (e.g., the main cause of failure for BHVs) is slow, gradual, and progressive [172], PHVs are exposed to sudden failure (catastrophic failure). Indeed, discontinuities and defects can be produced during handling/suturing polymeric materials, leading to rupture through crack propagation [175]. Resistance to tearing is therefore essential to evaluate the feasibility of PHVs [176]: it can be experimentally determined by measuring tear propagation strength and suture retention strength [175]. Many variables can affect the in vivo resistance to failure, both of mechanical and chemical origin; therefore, it is quite difficult to obtain a reliable estimation of tear onset and time from onset to breakage.
8. Hydrodynamic complications: Some problems with PHV function can be categorized as hydrodynamic issues. Poor valve design can lead to significant abnormalities in blood flow dynamics and/or damages of blood components; improper valve design can also result in the incomplete closure of the valve leaflets, leading to the backflow of blood or regurgitation.

## 10. Conclusions

In the quest for the ideal heart valve that can overcome the limitations of current bioprosthetic and mechanical valves, the next-generation solution must be integrated into the patient. This requires not only optimized fluid dynamics but also enhanced chemical interaction between the valve material and the biological environment. Additionally, such valves should be implanted through transcatheter techniques, such as TAVI, while ensuring low cost and widespread accessibility to make them available to a broader population [100].

PHVs should offer a promising alternative to traditional prosthetic devices, with significant potential to improve the quality of life for patients. In particular, they might benefit from the catheter-based implantation of PHVs. Despite the wide range of materials, manufacturing techniques, and valve designs presented in this work, the requirements for biostability and mechanical properties, the complexity of the valve model in an anisotropic environment, and the complicated structure and functioning of the native valve to be replaced, the research advancements and technological developments in this field are slow and challenging. However, what was thought impossible years ago has now become possible. Advancements in technology now allow us to overcome the limitations of first-generation polymers, which were the earliest synthetic materials used in biomedical applications. These polymers had a limited ability to withstand physiological conditions due to degradation and wear. Today, new polymers, which can be functionalized or combined with other materials, offer significantly enhanced mechanical and biological properties.

### 10.1. Future Directions

The clinical applications of PHVs will surely benefit from some improvements: among others, the biochemical functionalization of polymeric surfaces should favor the endothelization process, making the surface much more hemocompatible and mitigating the risk of blood clot formation [177]. Indeed, the in vivo endothelialization of PHVs has not been reported yet in the scientific literature, but various materials have been investigated [177].

Another strategy can be mentioned: One extensively studied surface modification involves the incorporation of polyethylene glycol (PEG) [178]: PEG-coated surfaces create tightly bound layers of water that prevent protein adsorption, significantly reducing thrombus formation. However, incorporating PEG into polymers has some drawbacks, such as triggering the complement pathway, and causing hypersensitivity reactions [179]. Other surface modifications, such as the incorporation of poly(vinyl chloride) (PVC), poly(2-ethyl-2-oxazoline), polyethylene oxide (PEO), titanium oxides/nitrides, polysulfone (PSF), and poly(ethylene), have been exploited to increase hemocompatibility but with limited success [177,180].

Advantageous cellular functions can also be promoted (and those unwanted, mitigated) by means of bioactive molecules (e.g., biologically active peptides, proteins, and growth factors) chemically grafted to the surface [181].

An example of a peptide suitable for such applications is Arg-Glu-Asp-Val (REDV), derived from fibronectin. This peptide selectively binds to α4β1 integrin, predominantly found on endothelial cells (ECs), and has been widely studied for its potential in modifying biomaterial surfaces to enhance endothelialization [182]. For instance, Butruk et al. developed a method to modify polyurethane (PU) surfaces with REDV-containing peptides, demonstrating improved EC adhesion and proliferation [183,184].

Moreover, a number of treatments can be applied to enhance blood compatibility and bactericidal activity by changing surface characteristics like roughness, wettability, and energy, for example, plasma treatment, ion implantation, and ultraviolet (UV) irradiation [185].

Another strategy was proposed by Benson et al.: they used a coating with two covalently linked hyperbranched polymer thin films, one adhering to the surface and one preventing protein adsorption. They showed promising results in terms of durability and cell adhesion [186].

In addition to these approaches, an emerging option for improving material biocompatibility involves the use of hybrid materials. These combine a polymeric component with a biologically derived material, leveraging the advantages of both to enhance mechanical and biological performance [187,188,189]. Other studies investigated the application of drug-releasing multilayer coatings to reduce the use of anticoagulation drugs [190], and hydrogel coatings that enhance hydrophilicity and blood compatibility, maintaining platelet resistance [191].

More traditional methods such as topographical modifications have to be cited: they create micro- or nano-scale patterns on the surface that are able to influence cell behavior and protein adsorption without adding chemical substances [180]. Altering surface roughness can affect blood cell adhesion [192], and surfaces with specific micro-structures similar to native tissues have shown higher super hydrophobicity and blood compatibility than smooth surfaces [193]. A recent study by Vigano et al. highlighted that surface nanotexture can minimize thrombogenicity, although further specific studies are needed [194].

Finally, we mention the exhaustive work published by Hofferberth et al. [195] that describes a biomimetic balloon-expandable heart valve prosthesis specifically conceived for the pediatric population. This valve is able to accommodate somatic growth and structural asymmetries within the heart. This prosthetic device was designed by taking inspiration from the human venous valve. Prototypes were fabricated from 0.1 mm thickness ePTFE leaflets handsewn to a laser-cut stainless-steel stent. Numerical simulations and in vitro and in vivo (animal model) experiments allowed them to validate the functional performance of the valve.

### 10.2. Is Clinical Translation near or Far?

Prosthetic heart valves are classified as Class III medical devices by the FDA; therefore, they require the highest level of regulatory examination before approval for the market by the competent authorities (e.g., the FDA in the US, notified bodies in the EU). For this purpose, the biggest challenge for PHVs is the successful demonstration of long-term durability in vivo: 10–15 years (or more) can be a reasonable timeframe for approval. Moreover, any new prosthetic device has to exhibit better performance compared to the existing ones and, in the specific case of PHVs, the mechanism of failure has to be precisely addressed. All these issues are particularly challenging!

The valve made of Hastalex has been thoroughly assessed in vitro and in vivo: it showed excellent features in terms of mechanical properties, hemocompatibility, and calcific resistance. Nevertheless, further in vivo experiments are mandatory “to estimate long-term biocompatibility and biostability of Hastalex as a heart valve prosthesis” [81]. So, the clinical translation of this valve seems to be not so close.

After extensive in vitro and in vivo evaluations, the TRIA valve (which is the one made of SiPUU) was implanted in one patient (first-in-human) with aortic valve disease at Beaumont Hospital (Royal Oak, MI, USA), as part of an FDA Early Feasibility Study (EFS). Subsequently, it was authorized to enter clinical trials. The TRIA valve met all of its primary endpoints at one year, including improvement in EOA, clinically significant improvement in the New York Heart Association (NYHA) class, and safety [87]. In 2021, Foldax announced the successful first-in-human use of the TRIA valve for the replacement of a diseased mitral valve as well: surgery was performed by David Heimansohn, at Ascension St. Vincent Hospital, Indianapolis, IN, (USA). The TRIA valve, which is currently considered investigational in the USA and is not yet commercially available, seems to be not far from receiving market authorization.

## Figures and Tables

**Table 1:** References cited per paper section.

Section	References
1. Introduction	[1,2,3,4,5]
2. Native heart valves	[6,7,8,9,10]
3. Valvular diseases	[11,12,13,14,15,16,17,18,19,20,21,22]
4. Current therapeutic approaches	[15,23,24,25,26,27,28,29,30,31,32,33,34,35,36,37]
5. Prosthetic heart valves	[38,39,40,41,42,43,44,45]
6. Polymeric heart valves	[46,47,48,49,50,51,52,53,54,55,56,57,58,59,60,61,62,63,64,65,66,67,68,69,70,71,72,73,74,75,76,77,78,79,80,81,82,83,84,85,86,87,88,89,90,91,92,93,94,95,96,97,98,99,100,101,102,103,104,105,106,107,108,109,110,111,112,113,114,115,116,117,118,119,120,121,122,123,124,125,126,127,128,129]
7. Manufacturing techniques	[48,50,51,71,74,78,80,118,123,130,131,132,133,134,135,136,137,138,139,140,141,142,143,144,145,146,147,148,149,150,151]
8. Overall consideration of valve design and geometry	[48,67,71,73,78,105,106,134,135,136,152,153,154,155,156,157,158,159,160,161,162,163,164,165,166]
9. Potential complications associated with the use of PHVs	[90,167,168,169,170,171,172,173,174,175]
10. Conclusions	[81,87,100,176,177,178,179,180,181,182,183,184,185,186,187,188,189,190,191,192,193,194,195]

**Table 2:** Evolution of polymeric heart valves over recent years.

Description	Reference
One of the first designs for PHVs, with cone-shaped leaflets and made of silicon	Roe et al. [48]
Polyurethane trileaflet valve: composed of three hemicylindrical leaflets, each with a 10 mm diameter with respect to the flow axis, and an orifice diameter of 23.5 mm	Wisman et al. [157]
Cylindrical trileaflet heart valve	Lockie et al. [158]
Polyurethane heart valve: in the closed position, the leaflet shape is characterized by a conic section: hyperbolic in the circumferential (x-y plane) and elliptical in the radial direction (x-z plane)	Mackay et al. [71]
Leaflet geometry defined by a (a) hyperboloid of revolution around an axis and (b) revolution around an axis of an arc subtending two straight lines	Jiang et al. [105]
PVA-BC heart valve: without gaps between the two adjacent leaflets and a middle orifice area < 5% of the total orifice area in the closed position	Mohammadi et al. [106]
SSAV prototype made of POSS-PCU with an internal diameter of 21 mm, external diameter of 22 mm, aortic protrusion of 14 mm, and total height of 20 mm	Rahmani et al. [78]
Valve design characterized by two curves (circumferential and radial), optimized to achieve the maximum EOA, minimal regurgitation, and less damage concentration in the leaflets	Gharaie and Morsi [163]

**Table 3:** Summary of the materials used for the production of prosthetic heart valves, processing methods, and general characteristics. * Abbreviations: A.M. = additive manufacturing, D.M. = dip molding, I.M. = injection molding, M.B. = melt blending, S.C. = solvent casting, E.S. = electrospinning. A green dot indicates the presence of the characteristic; a red dot, the absence; and a question mark indicates that the characteristic has not been mentioned or assessed.

	Material	Processing Methods *	In Vitro Test	In Vivo Test	Mechanical Performances	Hydrodinamic Performances	Chemical Stability	Calcification	Biocompatibility	Blood Compatibility
First generation of polymers	PDMS [46,47,49,52]	A.M., D.M.	●	●	●	●	●	?	●	●
	PDMS-PET [50]	I.M., D.M., A.M.	?	●	●	●	?	?	●	●
	PDMS-PP [52]	M.B., S.C., A.M.	?	●	●	●	●	?	●	●
	PDMS-PHMO [54]	D.M., S.C.	●	●	●	●	●	?	●	●
	PDMS + PU [55,56,57]	D.M., S.C., A.M.	●	●	●	?	●	●	●	●
	PDMS + PCU [58]	S.C., D.M., E.S.	●	●	●	?	●	?	●	?
	PDMS-SB-UU [59]	S.C., D.M., E.S.	●	●	?	?	●	?	●	●
	PTFE [60,61,62]	M.B., S.C., I.M., A.M.	?	●	●	●	●	●	●	●
	ePTFE [63]	D.M., I.M., A.M.	?	●	●	●	●	●	●	●
	PU [69]	D.M., I.M., A.M., E.S.	●	●	●	●	?	●	●	●
	PEU [72]		?	●		?	?	●	?	?
	PEUE [73]		?	●	●	?	?	?	?	?
	PCU [74]		●	●	●	●	●	●	●	●
Second generation of polymers	POSS-PCU [75,76]	I.M., D.M., A.M.	?	●	?	●	●	?	●	●
	FGO-PCU [81]	D.M., I.M., A.M., E.S.	?	●	●	●	●	●	?	?
	SiPUU [82]	I.M., D.M., A.M.	●	?	●	?	●	?	●	?
	SiPUU-PEEK-PTFE [86]		?	●	?	●	●	●	●	●
	SIBS [91]	I.M., D.M., A.M.	?	●	●	?	●	?	?	?
	SIBS-PP [92]		●	?	●	?	?	?	●	●
	SIBS + PET [94]		?	●	●	?	●	●	?	?
	xSIBS [93,94,96,97]		?	●	●	●	?	?	?	●
	SIBS + HDPET [94]		?	●	●	?	?	●	●	●
	SIBS + CNTs [99,101]		●	?	●	?	?	●	●	●
	PVA [103,104,105]	A.M., S.C.	●	?	●	●	?	?	?	?
	PVA-BC [106]		●	●	●	●	?	?	●	?
	GO-PVA [110,111]		?	?	●	●	?	?	?	?
	LLDPE [114]	I.M., A.M., D.M.	●	?	●	?	?	?	●	●
	HA-LLDPE [116,118]		●	?	●	?	?	?	●	●
	SEPS [119,120]	I.M., A.M., D.M.	●	?	●	?	●	?	●	●
	SEBS [121,122]		●	●	●	?	●	●	?	●
Other nanocomposites	MWCNTs-PA [124]	I.M., D.M., A.M., S.C.	●	?	●	?	?	?	●	?
	PLGA-CNF [125]	A.M., S.C., E.S.	●	?	?	?	?	?	●	?
	PPF [121]	A.M., S.C., I.M.	●	?	?	?	?	?	●	?
	PPFU [126]	A.M., S.C.	●	●	●	?	?	?	●	?
	SWNT-PPF [127]		?	●	?	?	●	?	●	?
	PU/GO-g-pMPC [128,129]		●	?	●	?	?	?	●	●
